# How *Listeria monocytogenes* Shapes Its Proteome in Response to Natural Antimicrobial Compounds

**DOI:** 10.3389/fmicb.2019.00437

**Published:** 2019-03-12

**Authors:** Rosalba Lanciotti, Giacomo Braschi, Francesca Patrignani, Marco Gobbetti, Maria De Angelis

**Affiliations:** ^1^Dipartmento di Scienze e Tecnologie Agro-Alimentari, Università degli Studi di Bologna, Bologna, Italy; ^2^Free University of Bozen, Bolzano, Italy; ^3^Dipartimento di Scienze del Suolo, della Pianta e degli Alimenti, Università di Bari Aldo Moro, Bari, Italy

**Keywords:** *Listeria monocytogenes*, proteomics, natural antimicrobials, stress, adaptation

## Abstract

The goal of this study was to investigate the adaptation of *L. monocytogenes* Scott A cells to treatments with sublethal doses of antimicrobials (ethanol, citral, carvacrol, E-2-hexenal and thyme essential oil). The survival of *L. monocytogenes* cells was not affected by the antimicrobials at the concentrations assayed, with the exception of ethanol (1% v/v) and thyme essential oil (100 mg/L), which decreased cell viability from 8.53 ± 0.36 to 7.20 ± 0.22 log CFU/mL (*P* = 0.04). We subsequently evaluated how *L. monocytogenes* regulates and shapes its proteome in response to antimicrobial compounds. Compared to the control cells grown under optimal conditions, *L. monocytogenes* treated for 1 h with the antimicrobial compounds showed increased or decreased (≥ or ≤2-fold, respectively, *P* < 0.05) levels of protein synthesis for 223 protein spots. As shown multivariate clustering analysis, the proteome profiles differed between treatments. Adaptation and shaping of proteomes mainly concerned cell cycle control, cell division, chromosome, motility and regulatory related proteins, carbohydrate, pyruvate, nucleotide and nitrogen metabolism, cofactors and vitamins and stress response with contrasting responses for different stresses. Ethanol, citral (85 mg/l) or (E)-2-hexenal (150 mg/L) adapted cells increased survival during acid stress imposed under model (BHI) and food-like systems.

## Introduction

*Listeria monocytogenes* is the etiologic agent of listeriosis, one of the major serious foodborne illnesses that occur worldwide (Swaminathan and Gerner-Smidt, 2007; Huang et al., 2014). Listeriosis results in the death of 20–30% of patients, primarily in elderly people, pregnant women, children or immune-compromised populations (Forsyth et al., 1998; Mead et al., 1999; Orsi et al., 2011). According to the European Union summary report on trends and sources of zoonoses, zoonotic agents and foodborne outbreaks (2017), there has been a significant increasing trend of confirmed listeriosis cases in the EU/EEA from 2008-to 2017 as well as during the last 5 years (period 2013–2017). In 2017, the European case fatality was 13.8% among the 1,633 confirmed cases with a known outcome, showing only a slight decrease compared to that observed in 2016.

*L. monocytogenes* is a saprophytic species that exhibits a high survival rate in food ecosystems, including fruits and vegetables (raw, cooked, and processed) (Hadjilouka et al., 2015), meat (Liu et al., 2014; Gouveia et al., 2016), milk (Sadeghi et al., 2016), and fish (Rocourt et al., 2003). *L. monocytogenes* is able to grow in different niches and has a strong ability to resist environmental and technological stresses, such as high/low temperatures and modified atmospheres. In addition, *L. monocytogenes* is able to adapt and survive to different stress conditions within the food chain (Gandhi and Chikindas, 2007; Chan and Wiedmann, 2008; Cacace et al., 2010; Dutta et al., 2013; NicAogáin and O’Byrne, 2016), including those used for food processing and storage (Gomes Neto et al., 2015). Consequently, *L. monocytogenes* constitutes a major risk to consumers and causes high economic losses (Cabrita et al., 2015). The European Commission (No 2073/2005) requires that the levels of *L. monocytogenes* in foods not intended for infants and hospitalized individuals and not representing an optimal medium for its growth must not exceed 100 CFU/g. In minimally processed fruits and vegetables, the use of chemicals (e.g., ozone, H_2_O_2_, organic acids, calcium-based solutions and peroxiacetic acids) as disinfectants is not sufficient to statistically decrease the survival of *L. monocytogenes* strains (Soliva-Fortuny and Martìn-Belloso, 2003; Patrignani et al., 2015). However, based on consumer concerns regarding chemical synthetic additives (Sivakumar and Bautista-Baños, 2014), one of the emerging strategies used to decrease the survival of *L. monocytogenes* in food products is the use of natural antimicrobial compounds alone or in combination with other mild chemicals (Kamdem et al., 2011; Ngang et al., 2014). Antimicrobial products produced by plants, such as essential oils (EOs) and six carbon atom aldehydes generated in the lipoxygenase pathway, play key roles in plant defense against microbial proliferation, with many of these products being generally recognized as safe (GRAS) and used to improve the sensory quality and shelf-life of fruits, vegetables, meat and dairy foods (Burt, 2004; Belletti et al., 2010). The antimicrobial properties of EOs are primarily related to presence of C10- and C15-terpenes with aromatic rings and phenolic-hydroxylic groups that can form hydrogen bonds with active sites of target enzymes (Picone et al., 2013). In addition, other compounds in EOs, such as alcohols, aldehydes and esters have antimicrobial effects. EOs affect bacterial and fungal viability differently depending on their composition and structural configuration as well as to the possible synergistic interactions among the components (Picone et al., 2013; Patrignani et al., 2015). Interestingly, some EOs have a wide spectrum of activity against pathogens, including *L. monocytogenes* (Oliveira et al., 2013; Patrignani et al., 2015). Some EOs from rosemary, thyme and oregano, such as citral (a mixture of monoterpene aldehydes present in geranial and neral), carvacrol, thymol, hexanal and trans-2-hexenal appear to be good natural antimicrobial candidates due to their inhibitory effects against bacteria and fungi in foods (Hao et al., 1998; Lanciotti et al., 2003, 2004; Burt, 2004; Cava-Roda et al., 2012; Ivanovic et al., 2012; Siroli et al., 2014; Patrignani et al., 2015). Compared to other common antimicrobial compounds and human pathogens, there is little information available regarding the use of natural antimicrobial products to decrease the biofilm formation, cell survival, and environmental adaptation of *L. monocytogenes* (Helke et al., 2017; Tracanna et al., 2017; Van Vuuren and Holl, 2017). Despite recent progress in elucidating the details of the *L. monocytogenes* genome, the mechanisms this bacterium adapts to natural antimicrobial compounds remains largely unclear (NicAogáin and O’Byrne, 2016; Braschi et al., 2018a,b). Studies on the adaptation of *L. monocytogenes* to natural antimicrobial compounds are crucial to highlight the relationship between stress and virulence and to optimize the protocols used for food production (Chaturongakul et al., 2008; Bowman et al., 2010; He et al., 2015).

Proteomic approaches linking genome and transcriptome to potential biological functions could highlight the molecular mechanisms of stress adaptations of *L. monocytogens* to natural antimicrobial compounds (Guevara et al., 2015). Accordingly, the goal of this study was to investigate the proteomic adaptation of *L. monocytogenes* Scott A cells during exposure to sub-lethal concentrations natural antimicrobials [ethanol, citral, carvacrol (E)-2-hexenal and thyme EO].

## Materials and Methods

### Bacterial Strains and Culture Conditions

*L. monocytogenes* Scott A was stored at -80°C for long-term preservation. To acclimatize the strain to the experimental conditions, 1 mL of the culture strain was inoculated into 9 mL of Brain Heart Infusion broth (BHI) (Thermo-fisher, Milano, Italy) and incubated for 24 h at 37°C. Subsequently, the cells were propagated at 37°C for 24 h in BHI broth using a 1% inoculum.

### Natural Antimicrobials

Citral, carvacrol and (E)-2-hexenal were obtained from Sigma-Aldrich (Milano, Italy), while thyme EO was purchased from Flora s.r.l. (Pisa, Italy). Stock solutions of the natural antimicrobials were diluted in absolute ethanol (Sigma-Aldrich, Milano, Italy) and stored for up to a month at 4°C until use.

### Antimicrobial Treatment Conditions

*L. monocytogenes* Scott A cells grown in BHI broth for 24 h at 37°C were inoculated (1% v/v) into 1000 mL of fresh BHI broth at a final density of 4 log CFU/mL. The cells were cultivated at the optimum growth temperature (37°C) until reaching the mid-exponential phase of growth (OD600 nm of 0.4). Next, the cells were harvested by centrifugation at 9,000 ×*g* for 10 min at 37°C and resuspended in 1000 mL of fresh BHI broth alone (untreated cells, control) or supplemented with antimicrobial compounds (treated cells). The concentrations and the conditions used were the same those reported in Braschi et al. (2018a,b). Antimicrobial treatments were performed using ethanol (final concentration in BHI broth of 1% v/v) alone or with citral (final concentrations in BHI broth of 85 or 125 mg/L, corresponding to 1/3 and 1/2 of the minimal inhibitory concentration – MIC), carvacrol (20, 35, or 50 mg/L, corresponding to 1/5, 1/3, and 1/2 of the MIC) (E)-2-hexenal (150, 250, or 400 mg/L, corresponding to 1/5, 1/3, and 1/2 of the MIC) and thyme essential oil (40, 70, or 100 mg/L, corresponding to 1/5, 1/3, and 1/2 of the MIC).

After incubating at 37°C for 1 h, the control and treated cells were harvested by centrifugation at 6000 rpm at 4°C for 10 min, and the pellet was used for total viable cell counts or stored at -80°C for protein extractions.

### Measurement of Antimicrobial Tolerance

The harvested cells were resuspended in fresh BHI broth, harvested by centrifugation at 9000 rpm at 4°C for 5 min and immediately resuspended in sterile physiological solution for plate count. Cell numbers were determined by plating on BHI agar medium, with the number of CFU determined after a 24 h incubation at 37°C. The number of surviving microorganisms was calculated as a percentage of the cell number at time zero. The tolerance factor (TF) corresponded to the ratio of the cells that survived the treatment to that of control cells.

### Protein Extraction and 2-DE Analyses

Cells in the exponential growth phase were harvested from BHI broth, washed in Tris-HCl 50 mM (pH 7.5) and used to generate proteins extracts as described by De Angelis et al. (2001). Equivalent amounts of total protein (60 μg for analytical runs or 200 μg for preparative runs for protein identification) were used for each electrophoretic run. The 2-DE was performed essentially as described by Görg et al. (1988) and Hochstrasser et al. (1988) using a Pharmacia 2-D-Electro Focusing (EF) system (GE Healthcare, Milano, Italy). Gels were stained using Brilliant Blue G-Colloidal Concentrate (Sigma) or an MS-compatible silver method. The protein maps were scanned using LabScan on an ImageScanner (GE Healthcare) and were analyzed using ImageMaster 2D Platinum v.6.0 (GE Healthcare). Three gels from three independent experiments were analyzed, and the spot intensities were normalized (De Angelis et al., 2001), with the spot quantification for each gel calculated as a relative volume (% vol) that corresponded to the volume of each spot divided by the total volume over the entire image. The comparison between different conditions for the amount of the same protein was carried out as the rate of the relative volume of the same spot found in control (untreated cells) and ethanol or other antimicrobials treated cells (Siragusa et al., 2014).

### Protein Identification

In-gel tryptic digestion was performed by washing gel pieces two times with 50% (v/v) aqueous acetonitrile containing 25 mM ammonium bicarbonate, after which they were washed once with acetonitrile and dried in a vacuum concentrator for 20 min. Next, sequencing-grade, modified porcine trypsin (Promega) was dissolved in 50 mM acetic acid supplied by the manufacturer and then was diluted fivefold with 25 mM ammonium bicarbonate to give a final trypsin concentration of 0.02 μg/μL. Next, the gel pieces were rehydrated by adding 10 μL of trypsin solution for 10 min, after which enough 25 mM ammonium bicarbonate solution was added to cover the gel pieces and the samples were incubated overnight at 37°C.

Subsequently, a 1 μL aliquot of each peptide mixture was applied to a ground steel MALDI target plate, which was followed immediately by the addition of an equal volume of a freshly prepared 5 mg/mL solution of 4-hydroxy-α-cyano-cinnamic acid (Sigma) in 50% aqueous (v/v) acetonitrile containing 0.1%, trifluoroacetic acid (v/v).

Positive-ion MALDI mass spectra were obtained using a Bruker Ultraflex III in reflectron mode, which was equipped with a Nd:YAG smart beam laser. MS spectra were acquired over a range of 800–4000 *m/z*. Final mass spectra were externally calibrated against an adjacent spot containing 6 peptides (des-Arg^1^-Bradykinin, 904.681; Angiotensin I, 1296.685; Glu^1^-Fibrinopeptide B, 1750.677; ACTH (1–17 clip), 2093.086; ACTH (18–39 clip), 2465.198; and ACTH (7–38 clip), 3657.929). Monoisotopic masses were obtained using a SNAP averaging algorithm (C 4.9384, N 1.3577, O 1.4773, S 0.0417, and H 7.7583) and an S/N threshold of 2.

For each spot, the ten strongest precursors were selected for MS/MS fragmentation, which was performed in LIFT mode without the introduction of a collision gas. The default calibration was used for to obtain MS/MS spectra, which were baseline-subtracted and smoothed (Savitsky-Golay, width 0.15 m/z, cycles 4). Monoisotopic peak detection was performed using a SNAP averaging algorithm (C 4.9384, N 1.3577, O 1.4773, S 0.0417, H 7.7583) with a minimum S/N of 6, and Bruker flexAnalysis (version 3.3) was used to perform spectral processing and to generate peak lists.

Tandem mass spectral data were used in database searches using a locally run copy of the Mascot program (Matrix Science Ltd., version 2.5.1) through the Bruker ProteinScape interface (version 2.1). The criteria specifications were as follows: Enzyme, Trypsin; Fixed modifications, Carbamidomethyl (C); Variable modifications, Oxidation (M) and Deamidated (NQ); Peptide tolerance, 100 ppm; MS/MS tolerance, 0.5 Da; and Instrument, MALDI-TOF-TOF. A minimum of two peptides with an ions score of at least 40 was required to get a reliable identification (Koskenniemi et al., 2009).

### Survival of *Listeria monocytogenes* to Acid Stress After Adaptation to Natural Antimicrobials

*L. monocytogenes* Scott A cells in the mid-exponential growth phase (OD = 0.4 at λ = 600 nm) were incubated for 1 h to sublethal concentrations of natural antimicrobials at 37 °C without stirring. Subsequently, the *L. monocytogenes* Scott A cells were inoculated at 6–7 log CFU/mL in BHI broth at different pH values (4.5 or 5.5). The growth capabilities and adaptation of the cells to acidic environments was evaluated over a 48 h incubation at 37°C by determining the optical density (OD) at 600 nm or counting CFUs on BHI agar-solidified medium. The same trials were also performed in a food model system consisting of a fruit-based beverage (mixture of rice drink and pear juice) at two pH values (4.5 and 5.5) that was obtained by modifying the ratio of rice drink and pear juice.

### Statistical Analyses and Bioinformatics

All experiments were performed in triplicate, and the data were subjected to a one-way ANOVA (SAS, 1985), with pairwise comparisons of treatment mean values performed using Tukey’s procedure at *P* < 0.05 using Statistica for Windows (Statistica 6.0 per Windows 1998). Principal component analysis (PCA) was performed suing Statistica for Windows and PermutmatrixEN to analyze the proteome profiles (De Angelis et al., 2008, 2015). Permutation analysis was performed using the relative volume of protein spots as found in each conditions (Siragusa et al., 2014). Changes in the protein amount (average of three replicates) are represented colorimetrically, with red and green indicating the highest and lowest values of the standardized data, respectively, for each protein of different conditions. All data were shown as a percentage of dissimilarity using Euclidean distance. To study changes in the expression of metabolic enzymes related to the adaptation to different antimicrobial products, all of the identified proteins were mapped to Kyoto Encyclopedia of Genes and Genomes pathways using both the enter gene ID and/or EC number functions^1^ (Bove et al., 2012). The comprehensive symbolic systems biology Pathway Tools (PT) software version 19.0 and the relative encompassed MetaCyc multiorganism database were used to reconstruct the metabolic pathways. The sample differences were normalized at the reaction, pathway and mega-pathway hierarchical levels. The use of custom scripts and manually checking steps allowed us to improve the functional characterization for the EC numbers, KEGG codes, and multiple subunits taking part in the same enzymatic reaction. To track the enzymes in our datasets, we used the REST-style KEGG API to link KEGG and MetaCyc pathways to EC numbers.

## Results

### Antimicrobial Stress Resistance

After reaching the mid-exponential phase, cells were exposed for 1 h to sublethal dose of antimicrobial compounds [ethanol, citral, carvacrol (E)-2-hexenal and thyme EO]. Ethanol was used at 1% (v/v) alone and was also used to convey the other antimicrobial compounds assayed. Citral was used at concentrations corresponding to 1/3 and 1/2 of the MIC. Carvacrol (E)-2-hexenal and thyme EO were used at concentrations corresponding to 1/5, 1/3, 1/2 of the MIC. According to observed MIC values, *L. monocytogenes* Scott A survival was unaffected by the antimicrobial treatments (data not shown), with the exception of cells treated with thyme EO (100 mg/L), which showed a decrease in cell viability from 8.53 ± 0.36 to 7.20 ± 0.22 log CFU/mL with a tolerance factor of 0.85 (*P* = 0.04).

### Proteomic Profiling

**FIGURE 1:**
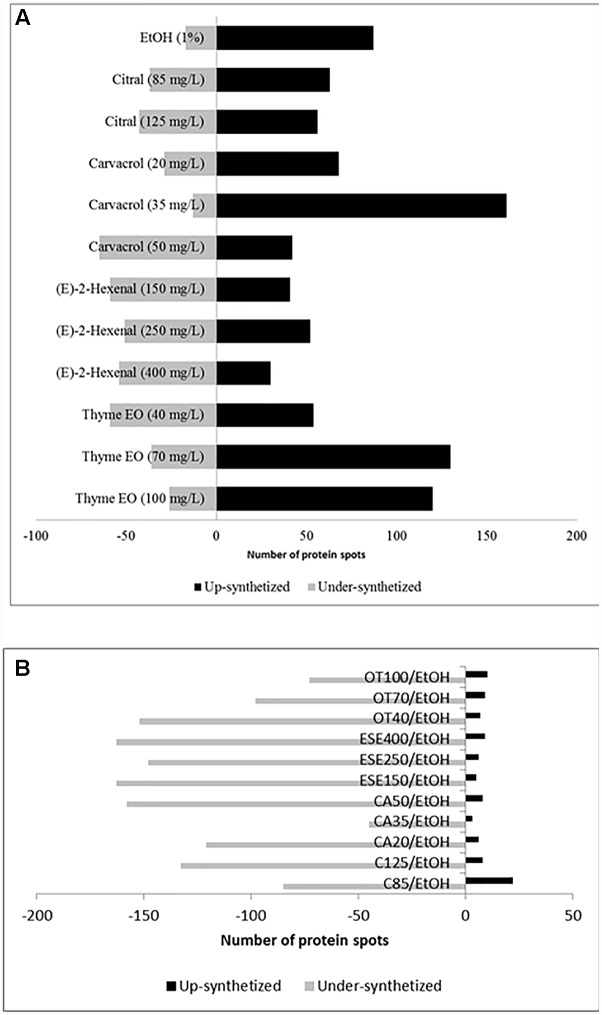
Number of protein spots showing increased and decreased (≥ or ≤of 2-fold, *P* < 0.05) levels of synthesis when *Listeria monocytogenes* Scott A cells were treated for 1 h at 37°C with antimicrobial compounds. C, untreated cells, control; EtOH, cells treated with ethanol (1% v/v) alone. Other antimicrobial compounds were: citral at 85 (C85) and 125 (C125) mg/L; carvacrol at 20 (CA20), 35 (CA35) or 50 (CA50); (E)-2-hexenal at 150 (ESE1), 250 (ESE2) or 400 (ESE4) mg/L; and Thyme essential oil at 40 (OT40), 70 (OT70), or 100 (OT100) mg/L. The comparison was performed by using untreated cells **(A)** or cells ethanol-treated cells **(B)**.

Compared to the control cells grown under optimal conditions, *L. monocytogenes* Scott A cells treated for 1 h with antimicrobial compounds showed increases or decreases (≥ or ≤ 2-fold, *P* < 0.05) in the levels of 223 protein spots (Figure 1A and Supplementary Figures 1–6 and Supplementary Table 1). In detail, ethanol-stressed cells exhibited increases in the levels of 87 protein spots compared to the control. In contrast, the relative amounts of 17 other protein spots were higher in control cells. The highest number of induced protein spots was observed for cells treated with thyme EO at 70 (130 spots) and 100 (120 spots) mg/L, as well as for cells treated with carvacrol at 35 mg/L (161 spots). Higher protein induction was observed in cells exposed to doses corresponding to 1/3 of the MIC values of citral, carvacrol (E)-2-hexenal and thyme EO. In contrast, the number of underexpressed protein spots was the highest using doses of antimicrobial products corresponding to 1/5 (E-2-hexenal and thyme essential oil) and/or 1/2 (citral and carvacrol) of the MICs. Proteins showing different relative amounts were analyzed by PCA. The proteome profiles differed between the treatments. The highest similarity among the different proteomes was observed for cells treated with citral (at 85 and 125 mg/L), or (E)-2-hexenal (150, 250 and 400 mg/L). Compared to the cells treated with ethanol, cells treated with other antimicrobials showed a strong reduction of the relative amount of various proteins (Figure 1B).

All of the proteins (223 spots) with relative abundances that were up- or downregulated were identified. Except for hypothetical or unknown proteins, the identified proteins were arranged by functional categories according to the KEGG database (Supplementary Table 2). The identified proteins were primarily involved in the following functional categories: (i) cell morphology and motility; (ii) ribosomal and regulation system proteins; (iii) carbohydrate transport and metabolism and energy production; (iv) nucleotide and nitrogen metabolism; (v) cofactor and vitamin metabolism; and (vi) general stress response. Numerous proteins were resolved as two or more spots on the 2-DE gels, possibly due to post-translational modifications (PTMs), protein cleavage or the presence of different isoforms (Ignatova et al., 2013). The identified proteins are described in detail in the following paragraphs.

### Cell Cycle Control, Cell Division, Chromosome, Motility, and Regulatory Related Proteins

**FIGURE 2:**
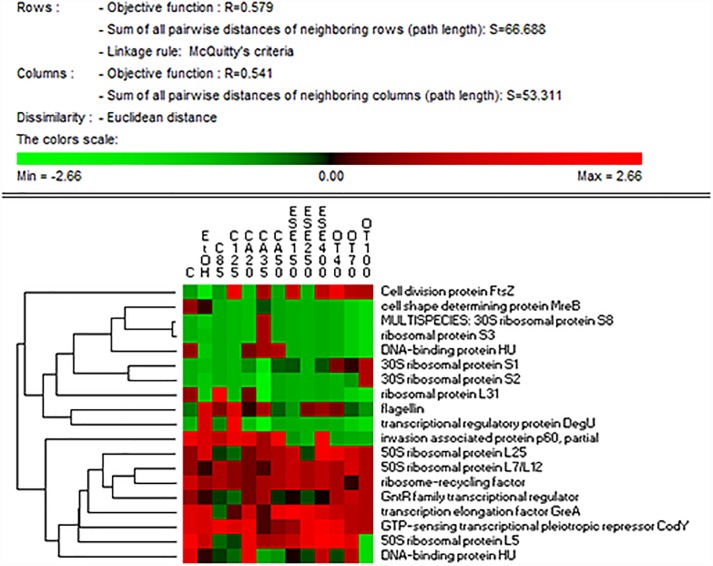
Heat map of the identified proteins involved in cell cycle control, cell division, chromosome, wall/membrane/envelope biogenesis, motility and regulatory mechanisms in cells of *Listeria monocytogenes* Scott A. Only protein spots showing different (≥ or ≤ of 2-fold, *P* < 0.05) relative amounts under stress conditions compared to untreated cells were showed. Permutation analysis was performed using the relative amount of protein spots as found in each condition. Changes in the protein amount (average of three replicates) are represented colorimetrically, with red and green indicating the highest and lowest values of the standardized data, respectively, for each protein of different conditions. All data were shown as a percentage of dissimilarity using Euclidean distance. C, untreated cells; EtOH, cells treated with ethanol (1% v/v) alone. Other antimicrobial compounds were: citral at 85 (C85) and 125 (C125) mg/L; carvacrol at 20 (CA20), 35 (CA35) or 50 (CA50); (E)-2-hexenal at 150 (ESE1), 250 (ESE2) or 400 (ESE4) mg/L; and thyme essential oil at 40 (OT40), 70 (OT70), or 100 (OT100) mg/L.

Compared to ethanol, citral, carvacrol (E)-2-hexenal or thyme EO treatments differently affected the relative amounts of FtsZ protein in cells, with the highest levels observed after citral (125 mg/L) and thyme EO (40 mg/L) exposure (Figure 2 and Supplementary Tables 1, 2). Compared to the ethanol, The cell shape-determining protein MreB decreased in treated cells, especially those exposed to thyme EO. Compared to the untreated cells, an increase (*P* < 0.05) in the level of flagellin A (FlaA) was observed in *L. monocytogenes* Scott A cells exposed to ethanol 1% (v/v). The addition of other antimicrobial compounds affected the inductive effect of ethanol on FlaA synthesis differently.

The highest levels of the 30S ribosomal proteins S1 and S2 were observed in cells treated with thyme EO at 100 mg/L. The treatment of *L. monocytogenes* Scott A cells with 35 mg/L carvacrol increased the levels of the 30S ribosomal proteins S3 and S8. In contrast, compared to the use of ethanol alone, the levels of the 30S ribosomal proteins L5 and L25 decreased in cells treated with citral and (E)-2-hexenal. The highest levels of ribosome recycling factor (Frr) were observed in cells treated with carvacrol (35 mg/L) or thyme EO at 100 mg/L.

The lowest levels of the transcription elongation factor GreA were observed in citral-treated cells. Compared to ethanol alone, the addition of other antimicrobials to the growth medium resulted in a reduction in the level of the protein DegU, with the exception of cells exposed to citral at 125 mg/L. The lowest levels of a GntR family transcriptional regulator were observed in cells treated with citral at 35 mg/L.

### Carbohydrate Metabolism

**FIGURE 3:**
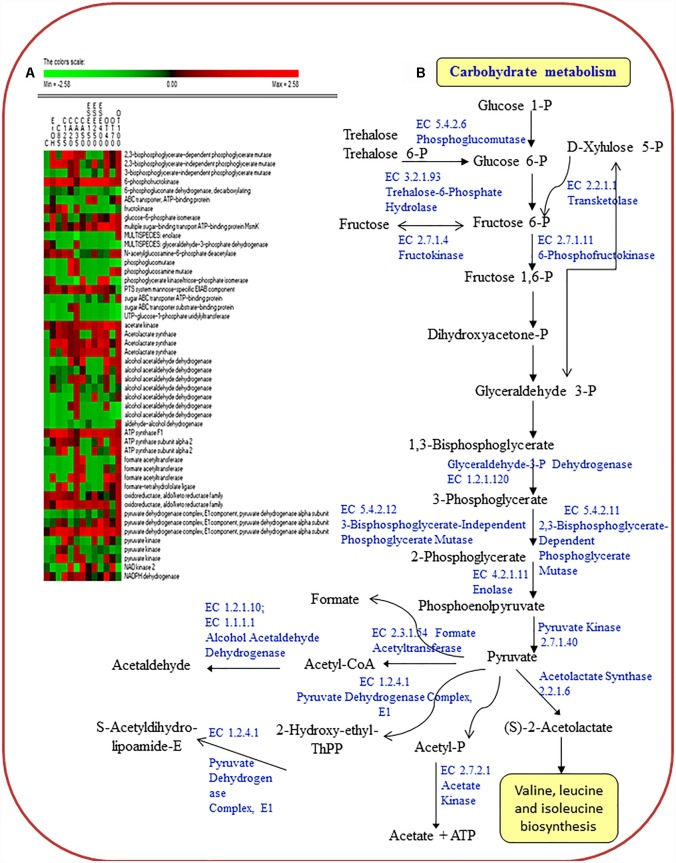
Heat map of the identified proteins **(A)** and reconstruction **(B)** of the carbohydrate metabolism in cells of *Listeria monocytogenes* Scott A. Only protein spots showing different (≥ or ≤ of 2-fold, *P* < 0.05) relative amounts under stress conditions compared to untreated cells were showed. Permutation analysis was performed using the relative amount of protein spots as found in each condition. Changes in the protein amount (average of three replicates) are represented colorimetrically, with red and green indicating the highest and lowest values of the standardized data, respectively, for each protein of different conditions. All data were shown as a percentage of dissimilarity using Euclidean distance. C, untreated cells; EtOH, cells treated with ethanol (1% v/v) alone. Other antimicrobial compounds were: citral at 85 (C85) and 125 (C125) mg/L; carvacrol at 20 (CA20), 35 (CA35) or 50 (CA50); (E)-2-hexenal at 150 (ESE1), 250 (ESE2) or 400 (ESE4) mg/L; and thyme essential oil at 40 (OT40), 70 (OT70) or 100 (OT100) mg/L.

The treatment of *L. monocytogenes* cells with ethanol and other antimicrobials had different effects on the level of proteins related to carbohydrate transport (sugar ABC transporter ATP-binding proteins; the multiple sugar-binding transport ATP-binding protein MsmK; and the PTS system mannose-specific EIIAB component ManX) (Figure 3).

Proteins associated with carbohydrate metabolism (3-bisphosphoglycerate-independent and dependent phosphoglycerate mutases, GpmA; glucose-6-phosphate isomerase, Pgi; phosphoglucosamine mutase, Pgm; pyruvate kinase, Pyk; and formate acetyltransferase, PflB) showed the highest relative abundance in *L. monocytogenes* Scott A cells treated with ethanol plus carvacrol (35 mg/L) and/or thyme EO (100 mg/L). With a few exceptions, carbohydrate metabolism proteins were underexpressed in cells treated with ethanol plus (E)-2-hexenal compared to ethanol alone.

### Nucleotide and Nitrogen Metabolism

**FIGURE 4:**
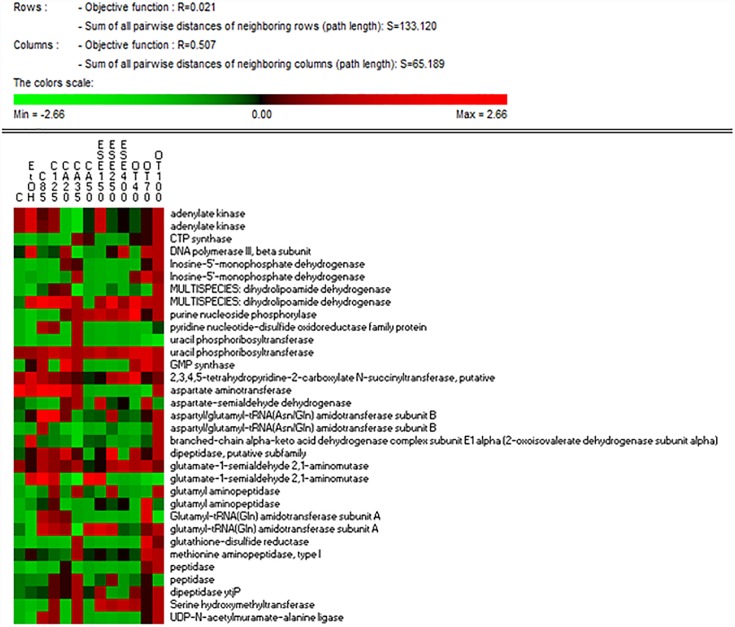
Heat map of the identified proteins involved in nucleotide and nitrogen metabolisms in cells of *Listeria monocytogenes* Scott A. Only protein spots showing different (≥ or ≤ of 2-fold, *P* < 0.05) relative amounts under stress conditions compared to untreated cells were showed. Permutation analysis was performed using the relative amount of protein spots as found in each condition. Changes in the protein amount (average of three replicates) are represented colorimetrically, with red and green indicating the highest and lowest values of the standardized data, respectively, for each protein of different conditions. All data were shown as a percentage of dissimilarity using Euclidean distance. C, untreated cells; EtOH, cells treated with ethanol (1% v/v) alone. Other antimicrobial compounds were: citral at 85 (C85) and 125 (C125) mg/L; carvacrol at 20 (CA20), 35 (CA35) or 50 (CA50); (E)-2-hexenal at 150 (ESE1), 250 (ESE2) or 400 (ESE4) mg/L; and thyme essential oil at 40 (OT40), 70 (OT70) or 100 (OT100) mg/L.

The treatment of *L. monocytogenes* cells with ethanol and other antimicrobials affected the levels of proteins related to nucleotide and nitrogen metabolism differently. The highest levels of GMP synthase [GuaA, EC:6.3.5.2], which is involved in purine metabolism (ko00230), were observed in cells exposed to ethanol alone for 1 h and especially in cells treated with ethanol plus citral (85 mg/L), carvacrol (20 or 35 mg/L) or thyme EO (Figure 4). The lowest levels of adenylate kinase [EC:2.7.4.3], which is involved in both purine and thiamine metabolism (ko00730), were observed in carvacrol-treated cells. The highest levels of CTP synthase, which is involved in pyrimidine metabolism (ko00240), were detected in cells treated with ethanol plus carvacrol (35 mg/L) and thyme EO (100 mg/L). The highest levels of uracil phosphoribosyltransferase [Upp, EC:2.4.2.9], which is involved in pyrimidine metabolism, were observed in cells exposed to carvacrol (at 20 mg/L). Purine-nucleoside phosphorylase [PpnP, EC:2.4.2.1], which is involved in purine, pyrimidine, nicotinate and nicotinamide metabolism (ko00760), was induced in cells treated with ethanol plus carvacrol (up to 35 mg/L) (E)-2-hexenal and thyme EO (up to 40 and 100 mg/L). The lowest levels of aspartate aminotransferase were observed in cells treated with carvacrol (50 mg/L) (E)-2-hexenal (all concentrations tested) or thyme EO at 40 and 70 mg/L. Except for aspartate aminotransferase, all of the other proteins related to the nitrogen metabolism were observed at higher relative amounts in cells exposed to the natural antimicrobials compared to the control ones cells. The highest relative amounts of peptidase enzymes were observed in *L. monocytogenes* Scott A cells treated with in ethanol plus carvacrol (20 and 35 mg/L) or thyme EO (70 and 100 mg/L).

### Cofactor and Vitamin Metabolism

Transketolase is an enzyme involved in the acyloin condensation reaction between C2 and C3 of pyruvate and glyceraldehyde 3-phosphate producing 1-deoxy-D-xylulose-5-phosphate (DXP) and plays a key role in the thiamine metabolism (ko00730). Transketolase was induced during ethanol stress exposure and remained at higher levels in all of the treated cells compared to the control cells (Supplementary Figure 7). Increased levels of formate-tetrahydrofolate ligase [EC:6.3.4.3], which is involved in the tetrahydrofolate interconversion of one carbon pool by folate (ko00670), were observed in cells treated with ethanol plus citral (all concentrations), carvacrol (35 mg/L) and thyme EO (all concentrations) compared to the control cells. Isochorismate synthase (MenF) and naphthoate synthase (MenB), which are involved in ubiquinone and other terpenoid-quinone biosynthesis (ko00130), were specifically induced in cells treated with ethanol plus carvacrol (35 mg/L) and thyme EO at 100 mg/L (MenF) and ethanol alone (MenB).

### Stress Response

The highest levels of the molecular chaperone DnaK were observed in the stressed cells with ethanol plus thyme EO at 100 mg/L (Supplementary Figure 8). Cells treated with ethanol plus carvacrol at 20 and 35 mg/L strongly increased GroEL, whereas the levels of GroES differed depending to the dose of antimicrobial used. Superoxide dismutase (Sod) was found at the maximum level in cells treated with ethanol plus carvacrol (35 mg/L) or thyme EO at 100 mg/L. Glutathione-disulfide reductase was specifically induced during cell exposure to ethanol plus carvacrol (35 mg/L) or thyme EO at 70 and 100 mg/L. The FeS assembly proteins SufB and SufD, which are associated with post-translational modification, protein turnover, and chaperones, were variably induced in cells exposed for 1 h to the natural antimicrobials tested. Proteins involved in fatty acid biosynthetic pathways and membrane repair (FabG and FabY) were induced under ethanol stress, especially in cells treated with ethanol plus citral (FabY) and ethanol plus citral (125 mg/L), carvacrol (35 and 50 mg/L), (E)-2-hexenal, or thyme EO (FabG).

### Acid Stress Resistance of Antimicrobial-Adapted *Listeria monocytogenes* Cells

**FIGURE 5:**
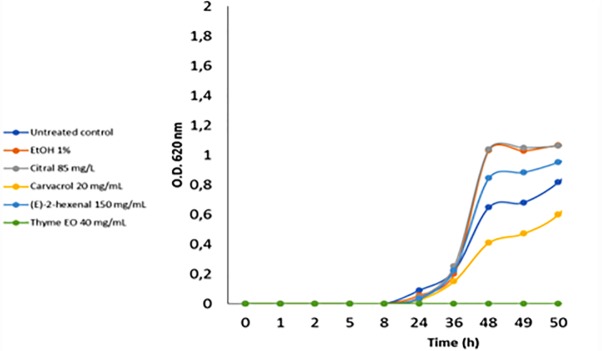
Increase of cell density of *Listeria monocytogenes* Scott A at pH 4.5 using as inoculum untreated and treated cells (for 1 h at 37°C) with antimicrobial compounds. EtOH, ethanol 1% (v/v); EO, essential oil.

The acid stress resistance of both control and treated *L. monocytogenes* cells was tested. Acid stress was imposed at pH 5.5 and 4.5 using a laboratory medium (BHI) and a food-like model (mixture of rice drink and pear juice). Compared to the control cells, cells treated with ethanol alone or ethanol plus citral at 85 mg/L increased (*P* < 0.05) the ability of *L. monocytogenes* Scott A cells to survive and grow in standard medium at pH 4.5 (Figure 5). Similar results were obtained using the food-like model system at pH 4.5 (data not shown). Treatment with 40 mg/L of thyme EO reduced the acid stress resistance of *L. monocytogenes* cells at pH 4.5 in both standard medium and the food-like model system.

## Discussion

Based on environmental conditions, bacteria modify cell morphology and protein synthesis to optimize growth, survival, and propagation (Woldemeskel and Goley, 2017). The short term (1 h) exposure to a sublethal level of ethanol reduced the levels of the cell division protein FtsZ, while the relative amount of the cell shape-determining protein MreB was unaffected. In rod-shaped bacilli, MreB controls cell length and promotes elongation of the cell wall, whereas FtsZ, a tubulin-like protein, is involved in the arrangement of the cell division septum and the resulting cell poles (Margolin, 2009). Both proteins are also involved in the synthesis of the glycan strands of peptidoglycan (Uehara and Park, 2008). Interestingly, ethanol stress was observed to downregulate the synthesis of FtsZ at the poles of other G+ bacteria (e.g., *Lactobacillus plantarum*) (Van Bokhorst-Van et al., 2011). Guevara et al. (2015) showed that the level of MreB increased during moderate heat, carvacrol and thymol treatments. Braschi et al. (2018b) investigated the effects of the natural antimicrobials assayed in this study on the morphological and physiological changes of *L. monocytogenes* and detected no significant effects on cell shape and dimension. In contrast, their flow cytometry results showed that cell membrane permeabilization, without a loss of esterase activity and cell membrane potential, was the common mechanism of action for all the antimicrobials assayed. Braschi et al. (2018a), studying the expression of genes whose products are involved in cell elongation and division (fusA and ftsE and ftsZ), showed significant underexpression of only the first two genes when *L. monocytogenes* was exposed for 1 h to trans (E)-2-hexenal and thyme EO. Previously, monoterpenes (e.g., citral, carvacrol, and thymol) were shown to primarily affect membrane structures, increasing membrane fluidity and permeability and leading to disturbances in the respiration chain, resulting in a subsequent dissipation of the proton-motive force (Lambert et al., 2001; Bakkali et al., 2008; Somolinos et al., 2008; Hyldgaard et al., 2012). In addition, Zengin and Baysal (2014), studying the effects of other terpenic natural antimicrobials against Gram-negative and Gram-positive pathogens, showed an increased cell constituent release and significant modification of the outer cell structures.

Patrignani et al. (2008) and Siroli et al. (2015) studied the effects of the exposure to natural antimicrobials assayed in this study at sublethal concentrations on the growth and membrane fatty acid composition of *L. monocytogenes*, demonstrating a marked increase of some membrane associated fatty acids, particularly unsaturated fatty acids, trans-isomers, and specific released free fatty acids. Recently, Braschi et al. (2018b) used flow cytometry to show an increased in cell membrane permeabilization without a loss of esterase activity and cell membrane potential with increasing citral, carvacrol and thyme EO concentrations. In contrast, (E)-2-hexenal did not significantly affect the measured physiological properties of *L. monocytogenes* Scott A.

Previously, *L. monocytogenes* strains were shown to regulate flagellar motility according to temperature (Cordero et al., 2016) and salt stresses (Durack et al., 2013). First, sublethal levels of natural antimicrobials were shown to affect the level of flagellin (FlaA) in a dose-dependent manner. Except for (E)-2-hexenal, the highest concentrations of natural antimicrobials considered did not significantly increase the levels of FlaA. The decreased level of flagellar proteins observed in most stressful conditions could be related to an energy saving mechanism under very stringent and harsh conditions, as previously observed in numerous studies for a variety of stresses (low temperature and osmotic stress) (Shen and Higgins, 2006; Hingston et al., 2015). However, persistence and the ability to form biofilms were not decreased in mutant strains with some deleted flagellar genes (Todhanakasem and Young, 2008). Overall, adaptation to different antimicrobials leads to modification of the amount of some ribosomal proteins involved in translation, ribosomal structure and biogenesis (30S ribosomal proteins S1, S2, S3, and S8 and 50S ribosomal proteins L5, L7/L12, L25, and L31). Previously, *L. monocytogenes* cells were shown to decrease the level of S1 protein under combined stresses (low pH, high salinity and low temperature) (He et al., 2015). Guevara et al. (2015) showed that the level of L7/L12 increased during moderate heat, carvacrol and thymol treatments. *L. monocytogenes* cells exhibited increased levels of some ribosomal proteins (S1 and L25) when exposed to ethanol alone. In the genus *Bacillus*, L25 (Ctc) works as general stress protein, showing a σ^B^-dependent promoter, and it was induced during osmotic, heat, oxidative and starvation stresses (Hecker and Volker, 1998). In *L. monocytogenes*, the expression of Ctc is dependent on σ^B^, an alternative factor encoded by sigB (Cacace et al., 2010). The transcription of σ^B^-dependent genes, which encode proteins related to transport, general stress response and metabolism, increased in *L. monocytogenes* under environmental stresses (Chan and Wiedmann, 2008). Ctc protein synthesis was observed to be upregulated in *L. monocytogenes* cells under osmotic stress (Gardan et al., 2003) and cold adaptation (Cacace et al., 2010). Consequently, ethanol-stressed cells could better survive during osmotic stress. In contrast, *L. monocytogenes* cells treated with a sub-lethal dose of (E)-2-hexenal, and especially, citral, exhibited inhibited Ctc synthesis, which could cause a decrease in survival under osmotic and cold stresses (Gardan et al., 2003; Cacace et al., 2010).

*L. monocytogenes* cells treated with specific concentrations of ethanol (1%) and carvacrol (35 mg/L) or thyme EO (100 mg/L) exhibited increased levels of ribosome recycling factor. Although this factor is reported to play a key role in bacterial growth, ribosomes can be regarded as the main cellular target for antibiotics, and microorganisms generally have numerous adaptation responses involved in increasing their resistance (Wilson, 2014). Based on the number of ribosomal proteins, a ribosomal response to antimicrobial treatments with ethanol and citral, carvacrol or thyme EO could also occur in *L. monocytogenes* Scott A.

As observed in other sequenced genomes, *L. monocytogenes* harbors regulatory proteins that play important roles in cell adaptation to different niches. First, this study showed that natural antimicrobials differently affected the level of synthesis of proteins involved in transcription (the GTP-sensing transcriptional pleiotropic repressor CodY, a GntR family transcriptional regulator, the transcription elongation factor GreA and the transcriptional regulatory protein DegU). CodY is a major cellular global regulator, serving as a repressor and activator of metabolic and virulence genes in G+ bacteria. Recently, CodY was shown to serve as a repressor or an activator of different genes regulating carbon and nitrogen metabolism, bacterial motility, stress related and virulence functions and metabolic adaptations in *L. monocytogenes* strains (Lobel and Herskovits, 2016). Interestingly, specific combinations of ethanol and carvacrol or (E)-2-hexenal reduced the relative abundance of CodY. In addition, cells treated with ethanol and citral were inhibited in the synthesis of the transcription elongation factor GreA. *L. monocytogenes* strains were previously observed to exhibit increased levels of GreA under heat and antimicrobial adaptation (55°C, 0.3 mM carvacrol and 0.3 mM thymol for 30 min) (Guevara et al., 2015) during treatment with lactic acid (Omori et al., 2017). GreA is an essential factor in the RNA polymerase elongation complex and protects proteins against aggregation. The overexpression of GreA was observed to increase bacterial resistance to heat and oxidative stress (Li et al., 2012; Omori et al., 2017). Consequently, cells treated with ethanol and citral, which showed the lowest level of GreA, could decrease the adaptability to heat and oxidative stress. Ethanol strongly increased the levels of the transcriptional regulatory protein DegU, which plays a role in motility, chemotaxis, biofilm formation and virulence in *L. monocytogenes* (Gueriri et al., 2008). The inductive effect of ethanol was reduced by adding carvacrol, (E)-2-hexenal and thyme EO due to the modulation of DegU phosphorylation by acetyl phosphate. The use of ethanol alone, and especially ethanol plus carvacrol at 35 mg/l or thyme EO at 100 mg/L, significantly increased the relative amounts of several proteins related to carbohydrate transport and metabolism. Some of these proteins (the PTS mannose transporter subunit IIAB, glyceraldehyde-3-phosphate dehydrogenase, bisphosphoglycerate-independent phosphoglycerate mutase and triosephosphate isomerase) were reported to be overexpressed in *L. monocytogenes CECT 4031* cells adapted at 55°C alone or in the presence of EOs (55°C and 0.3 mM carvacrol and 0.3 mM thymol) for 30 min (Guevara et al., 2015). The overexpression of proteins involved in carbohydrate transport and metabolism could be useful to compensate for partially impaired energy generation caused by antimicrobials interacting with the bacterial cytoplasmic membrane. Previously, the relative amount of enzymes related to carbohydrate metabolism in *L. monocytogenes* were shown to be affected during a redox shock (Ignatova et al., 2013). When natural antimicrobial compounds were added to ethanol-containing medium, *L. monocytogenes* Scott A adapted the synthesis of carbohydrate related proteins depending on the type and the specific concentrations of the natural antimicrobials used. Overall, carvacrol at 35 mg/L and thyme EO at 100 mg/L produced the highest induction in protein synthesis compared to citral, and especially (E)-2-hexenal. Redox shock also alters the pI or Mr of proteins, resulting multiple spots for the same protein. We observed that antimicrobial treatments drive the PTMs of several proteins. Horizontal spots (e.g., alcohol acetaldehyde dehydrogenase, formate acetyltransferase, and pyruvate kinase) can be attributed to PTMs such as phosphorylation, which play an important role in signal transduction and in the regulation of enzyme activities in bacteria (Ignatova et al., 2013). Several phosphorylated glycolytic enzymes are “moonlighting” proteins localized to the cell wall that interact with host components, increasing the adhesion and biofilm formation.

Stress proteins, such as glutathione-disulfide reductase, were induced in the cells treated with carvacrol at 35 mg/L and thyme EO at 100 mg/L and play a key role in maintaining the low intracellular redox potential required to have such molecules in their reduced form. Other proteins that were induced in the antimicrobial treatments, such as DnaK, are able to protect microbial cells during environmental stresses such as heat, acid, osmotic conditions. Kamdem et al. (2011) studied the effects of sublethal concentrations of carvacrol, (E)-2-hexenal, and citral on the heat resistance of *L. monocytogenes* 56LY. They demonstrated that the sublethal concentrations of the antimicrobial compounds used [i.e., 50 mg/L citral, 65 mg/L (E)-2-hexenal, and 30 mg/L carvacrol] did not prevent the growth of *L. monocytogenes* at 37°C but did enhance its inactivation. Between 55 and 63°C, the presence of the aroma compounds reduced by the time needed for a 5-log reduction of the microbial counts of the pathogenic species considered by approximately two-thirds. On the other hand, the effect of the increasing temperature on the toxicity of ethanol, essential oils and their components is well known (Lanciotti and Guerzoni, 1993; Guerzoni et al., 1994; Gardini et al., 1997; Lanciotti et al., 2004; Ndagijimana et al., 2004; Belletti et al., 2007; Patrignani et al., 2008, 2015). In fact, their antimicrobial activities are dependent on their logP, which enhances the partitioning of the molecules into the microbial membranes.

Compared to other stresses, few data are available about the cross-protection between antimicrobial adaptation and acid stress resistance in *L. monocytogenes*. Ethanol alone, ethanol plus citral or (E)-2-hexenal increased the ability of *L. monocytogenes* Scott A to survive and grow at pH 4.5. Interestingly, treatment with thyme EO reduced the acid stress resistance of *L. monocytogenes* Scott A also under the food-like system. The different acid resistance induced by antimicrobials could be related to the proteome plasticity of *L. monocytogenes* that improve the cell survival to specific environmental conditions.

The results of this study highlighted the cell adaptation of *L. monocytogenes* Scott A during treatments with sub-lethal doses of antimicrobials (ethanol, citral, carvacrol, E-2-hexenal, and thyme essential oil). Adaptation and shaping of proteomes primarily concerned cell cycle control, cell division, chromosome, motility and regulatory related proteins, carbohydrate, pyruvate, nucleotide and nitrogen metabolism, cofactors and vitamins and stress response with contrasting responses for different stresses. Cells adapted to ethanol, citral (85 mg/l) or (E)-2-hexenal (150 mg/L) exhibited increased survival during acid stress imposed under model (BHI) and food-like systems. A detailed understanding of the effects of natural antimicrobials on the microbial cell physiology and virulence is needed for their exploitation in the food industries as alternatives to traditional preservatives. The findings of this study can favor the use of natural antimicrobials for *L. monocytogenes* in the food industry as alternative preservatives to improve food safety. A full understanding of adaptive and survival strategies of pathogenic species such as L. monocytogenes should combine gene expression studies with proteome and phenotypic shifts. Otherwise, the fate of pathogens can be verified/and or predicted in real foods only by studying the proteome reprogramming and phenotypic switching associated with gene expression shift due to stress exposure. To improve food safety, a holistic approach is needed due to the incomplete information provided by individual analytical approaches. In contrast, the correlation between the genotypic, proteome, and phenotypic data is pivotal to fully understanding the activities and biological functions of pathogens under harsh conditions (i.e., those characterizing foods during storage and human gut). In this framework, this study determined the proteome shift in relation to the exposure to the natural antimicrobials considered to aid in controlling *L. monocytogenes* in foods to improve their safety.

## Data Availability

The raw data supporting the conclusions of this manuscript will be made available by the authors, without undue reservation, to any qualified researcher.

## Author Contributions

RL ideated the study and revised the manuscript. GB and FP performed all the experimental phases. MG discussed the results and revised the manuscript. MD directed the experimental phases the study and wrote the manuscript.

## Conflict of Interest Statement

The authors declare that the research was conducted in the absence of any commercial or financial relationships that could be construed as a potential conflict of interest.

## Footnotes

1 www.genome.jp/kegg/pathway.html

## Abbreviations

BHI: brain heart infusion broth
DXP: 1-deoxy-D-xylulose-5-phosphate
EO: essential oil
TF: tolerance factor
