# Supplementary material for: How Listeria monocytogenes Shapes Its Proteome in Response to Natural Antimicrobial Compounds
Source: Front Microbiol. 2019 Mar 12;10:437. doi: 10.3389/fmicb.2019.00437 (PMC6423498; doi:10.3389/fmicb.2019.00437)
Supplement: Supplementary file 2 [file Table_2.docx]

**Table S2.** Identified proteins synthesized by *Listeria monocytogenes* Scott A cells grown on BHI broth until the middle exponential phase of growth (OD= 0.4, λ=600 nm) was reached. Peptides were identified using MS/MS ion searches of the Mascot search engine (Mascot software v.2.0) and non-redundant NCBI database. Confidence in peptide identification was assessed by Mascot sequence assignment score and visual inspection of the MS/MS spectra. Both the maximum precursor ion mass tolerance and the MS/MS fragment ion mass tolerance were 0.2 Da. A peptide charge state of +1, +2 and +3 was used. A minimum of two peptides with an ions score of at least 40 was required to get a reliable identification.

| SPOT | PROTEIN | Microrganism | MR | PI | SCORE | COVERAGE (%) | Peptides |
| --- | --- | --- | --- | --- | --- | --- | --- |
| 2 | Alcohol Acetaldehyde Dehydrogenase | L. monocytogenes | 95036 | 6.58 | 387 | 10 | 7(7) |
| 3 | Alcohol Acetaldehyde Dehydrogenase | L. monocytogenes | 95036 | 6.58 | 640 | 11 | 8(8) |
| 4 | Alcohol Acetaldehyde Dehydrogenase | L. monocytogenes | 95036 | 6.58 | 633 | 11 | 8(8) |
| 5 | Alcohol Acetaldehyde Dehydrogenase | L. monocytogenes | 95036 | 6.58 | 491 | 18 | 6(6) |
| 7 | Alcohol Acetaldehyde Dehydrogenase | L. monocytogenes | 95036 | 6.58 | 609 | 10 | 8(8) |
| 8 | Molecular Chaperone Dnak | L. monocytogenes | 66092 | 4.57 | 623 | 12 | 6(6) |
| 12 | Transketolase | L. monocytogenes | 71831 | 5.11 | 393 | 10 | 6(6) |
| 13 | Transketolase | L. monocytogenes | 71854 | 5.11 | 384 | 18 | 5(5) |
| 15 | Formate Acetyltransferase | L. monocytogenes HCC23 | 84046 | 5.49 | 661 | 16 | 9(9) |
| 16 | Formate Acetyltransferase | L. monocytogenes HCC23 | 84046 | 5.49 | 585 | 14 | 8(8) |
| 17 | Formate Acetyltransferase | L. monocytogenes HCC23 | 84046 | 5.49 | 638 | 16 | 9(9) |
| 20 | Phosphoglucomutase | L. monocytogenes | 64232 | 5.03 | 222 | 18 | 4(4) |
| 21 | Chaperone Protein GroEL | L. monocytogenes str. 1/2a F6854 | 47082 | 4.64 | 779 | 22 | 7(7) |
| 22 | Pyruvate Kinase | L. monocytogenes serotype 4b str. H7858 | 59821 | 5.38 | 812 | 19 | 8(8) |
| 23 | Pyruvate Kinase | L. monocytogenes serotype 4b str. H7858 | 59821 | 5.38 | 826 | 19 | 8(8) |
| 24 | Pyruvate Kinase | L. monocytogenes serotype 4b str. H7858 | 59821 | 5.38 | 808 | 21 | 8(8) |
| 25 | Multispecies: Fe-S Cluster Assembly Protein Sufb | L. | 52711 | 4.87 | 366 | 12 | 5(5) |
| 26 | Multispecies: Fe-S Cluster Assembly Protein Sufb | L. | 52711 | 4.87 | 299 | 19 | 6(6) |
| 27 | Gmp Synthase | L. monocytogenes L99 | 58238 | 5.02 | 700 | 20 | 9(9) |
| 28 | Trehalose-6-Phosphate Hydrolase | L. monocytogenes | 62040 | 4.86 | 534 | 14 | 6(6) |
| 29 | Trehalose-6-Phosphate Hydrolase | L. monocytogenes | 63746 | 5.02 | 698 | 18 | 7(7) |
| 30 | Trehalose-6-Phosphate Hydrolase | L. monocytogenes | 62040 | 4.86 | 397 | 11 | 5(5) |
| 31 | Acetolactate Synthase | L. monocytogenes serotype 4b str. LL195 | 61991 | 5.47 | 511 | 14 | 7(7) |
| 32 | Acetolactate Synthase | L. monocytogenes serotype 4b str. LL195 | 61991 | 5.47 | 530 | 15 | 8(8) |
| 33 | Acetolactate Synthase | L. monocytogenes serotype 4b str. LL195 | 61991 | 5.47 | 596 | 17 | 9(9) |
| 34 | Ctp Synthase | L. monocytogenes serotype 4b str. LL195 | 62694 | 5.46 | 393 | 12 | 7(7) |
| 35 | Invasion Associated Protein P60, Partial | L. monocytogenes | 47381 | 9.15 | 758 | 25 | 9(9) |
| 36 | 3-Bisphosphoglycerate-Independent Phosphoglycerate Mutase | L. monocytogenes | 56063 | 5.10 | 434 | 11 | 4(4) |
| 37 | 2,3-Bisphosphoglycerate-Independent Phosphoglycerate Mutase | L. monocytogenes | 56130 | 5.18 | 574 | 18 | 8(8) |
| 38 | Formate--Tetrahydrofolate Ligase | L. monocytogenes 07PF0776 | 59945 | 5.28 | 366 | 19 | 4(4) |
| 39 | Heme Abc Transporter Atp-Binding Protein | L. monocytogenes | 56649 | 5.78 | 362 | 11 | 5(5) |
| 40 | Heme Abc Transporter Atp-Binding Protein | L. monocytogenes | 56649 | 5.78 | 424 | 12 | 6(6) |
| 41 | Inosine-5'-Monophosphate Dehydrogenase | L. monocytogenes serotype 4b str. LL195 | 55258 | 7.18 | 486 | 15 | 6(6) |
| 42 | Inosine-5'-Monophosphate Dehydrogenase | L. monocytogenes serotype 4b str. LL195 | 55258 | 7.18 | 440 | 14 | 6(6) |
| 43 | Hypothetical Protein Lm5578_1722 | L. monocytogenes 08-5578 | 49331 | 6.10 | 449 | 18 | 7(7) |
| 44 | Alcohol Acetaldehyde Dehydrogenase | L. monocytogenes | 95036 | 6.58 | 132 | 12 | 2(2) |
| 45 | Cell Division Protein Ftsz | L. monocytogenes serotype 4b str. LL195 | 43612 | 4.88 | 405 | 14 | 6(6) |
| 46 | Dipeptidase, Putative Subfamily | L. monocytogenes str. 1/2a F6854 | 27710 | 5.50 | 504 | 25 | 6(6) |
| 47 | Putative Dipeptidase Ytjp | L. monocytogenes serotype 4b str. LL195 | 53330 | 4.95 | 753 | 19 | 8(8) |
| 49 | Multispecies: Dihydrolipoamide Dehydrogenase | L. monocytogenes | 49571 | 5.24 | 377 | 12 | 4(4) |
| 50 | Multispecies: Dihydrolipoamide Dehydrogenase | L. monocytogenes | 49571 | 5.24 | 479 | 15 | 6(6) |
| 53 | 30s Ribosomal Protein S1 | L. monocytogenes | 41317 | 4.50 | 791 | 29 | 9(9) |
| 54 | Atp Synthase F1 | L. monocytogenes J2818 | 52408 | 4.80 | 1191 | 36 | 10(10) |
| 55 | Phosphoglucosamine Mutase | L. monocytogenes | 48600 | 4.72 | 449 | 16 | 7(7) |
| 56 | Glutamyl-Trna(Gln) Amidotransferase Subunit A | L. monocytogenes serotype 4b str. LL195 | 53586 | 5.03 | 281 | 17 | 3(3) |
| 57 | Glutamyl-Trna(Gln) Amidotransferase Subunit A | L. monocytogenes | 52434 | 4.92 | 327 | 19 | 4(4) |
| 58 | 6-Phosphogluconate Dehydrogenase, Decarboxylating | L. monocytogenes str. 4b H7858 | 51107 | 5.10 | 431 | 16 | 6(6) |
|  | Glutamate Decarboxylase | L. monocytogenes FSL F2-208 | 46398 | 5.13 | 165 | 15 | 2(2) |
| 59 | Aspartyl/Glutamyl-Trna(Asn/Gln) Amidotransferase Subunit B | L. monocytogenes FSL F2-208 | 53287 | 5.42 | 394 | 12 | 5(5) |
| 60 | Atp Synthase Subunit Alpha 2 | L. monocytogenes serotype 4b str. LL195 | 55447 | 5.34 | 625 | 15 | 6(6) |
| 61 | Aspartyl/Glutamyl-Trna(Asn/Gln) Amidotransferase Subunit B | L. monocytogenes FSL F2-208 | 53287 | 5.42 | 444 | 12 | 5(5) |
| 62 | Atp Synthase Subunit Alpha 2 | L. monocytogenes serotype 4b str. LL195 | 55447 | 5.34 | 693 | 15 | 7(7) |
| 64 | Gid Protein | L. monocytogenes J2818 | 40314 | 5.25 | 97 | 16 | 3(3) |
| 66 | Isochorismate Synthase | L. monocytogenes | 51763 | 5.61 | 164 | 19 | 4(4) |
| 67 | Glutathione-Disulfide Reductase | L. monocytogenes FSL F2-208 | 49277 | 5.66 | 79 | 17 | 2(2) |
| 68 | Alcohol Acetaldehyde Dehydrogenase | L. monocytogenes | 95036 | 6.58 | 36 | 10 | 2(2) |
| 71 | Carnitine Transport Atp-Binding Protein Opuca | L. monocytogenes serotype 4b str. LL195 | 51456 | 5.94 | 132 | 18 | 3(3) |
| 72 | Beta-Ketoacyl-Acyl-Carrier-Protein Synthase Ii | L. monocytogenes FSL F2-208 | 18913 | 5.17 | 40 | 12 | 2(2) |
| 73 | Tellurite Resistance Protein Tela | L. monocytogenes | 45464 | 5.32 | 54 | 15 | 2(2) |
| 74 | Hypothetical Protein Lmosa_4530 | L. monocytogenes str. Scott A | 47451 | 5.44 | 110 | 17 | 2(2) |
| 76 | Fes Assembly Protein Sufd | L. monocytogenes serotype 4b str. F2365 | 47587 | 5.61 | 59 | 15 | 2(2) |
| 77 | Fes Assembly Protein Sufd | L. monocytogenes serotype 4b str. F2365 | 47587 | 5.61 | 40 | 10 | 2(2) |
| 78 | Pyridine Nucleotide-Disulfide Oxidoreductase Family Protein | L. monocytogenes FSL F2-208 | 48588 | 5.39 | 54 | 13 | 2(2) |
| 79 | Udp-N-Acetylmuramate--Alanine Ligase | L. monocytogenes FSL F2-208 | 42673 | 5.55 | 73 | 15 | 2(2) |
| 80 | Aldehyde-Alcohol Dehydrogenase | L. monocytogenes str. 1/2a F6854 | 68004 | 6.31 | 171 | 17 | 4(4) |
| 81 | Pantothenate Metabolism Flavoprotein Homolog | L. monocytogenes | 43426 | 6.10 | 31 | 10 | 2(2) |
| 82 | Sugar Abc Transporter Substrate-Binding Protein | L. monocytogenes | 46603 | 4.60 | 267 | 15 | 5(5) |
| 83 | Glutamate-1-Semialdehyde 2,1-Aminomutase | L. monocytogenes | 46658 | 5.47 | 65 | 19 | 2(2) |
| 85 | Acetate Kinase | L. monocytogenes str. 1/2a F6854 | 44794 | 5.33 | 241 | 15 | 5(5) |
| 86 | Serine Hydroxymethyltransferase | L. monocytogenes serotype 4b str. LL195 | 47287 | 5.76 | 72 | 13 | 2(2) |
| 88 | Dna Polymerase Iii, Beta Subunit | L. monocytogenes FSL F2-208 | 28610 | 5.00 | 75 | 19 | 2(2) |
| 89 | Branched-Chain Alpha-Keto Acid Dehydrogenase Complex Subunit E1 Alpha | L. monocytogenes | 36603 | 4.96 | 66 | 14 | 2(2) |
| 92 | Peptidase | L. monocytogenes FSL R2-503 | 40757 | 5.07 | 30 | 18 | 2(2) |
| 94 | Peptidase | L. monocytogenes FSL R2-503 | 40757 | 5.07 | 46 | 12 | 2(2) |
| 96 | Glutamate-1-Semialdehyde 2,1-Aminomutase | L. monocytogenes | 46658 | 5.47 | 97 | 16 | 3(3) |
| 97 | Aspartate Aminotransferase | L. monocytogenes FSL J2-071 | 24034 | 5.21 | 114 | 13 | 2(2) |
| 100 | Conserved Hypothetical Protein | L. monocytogenes FSL J2-071 | 36334 | 6.23 | 159 | 18 | 3(3) |
| 101 | N-Acetylglucosamine-6-Phosphate Deacetylase | L. monocytogenes FSL F2-208 | 41619 | 5.43 | 98 | 13 | 3(3) |
| 102 | Sugar Abc Transporter Atp-Binding Protein | L. monocytogenes | 41179 | 5.82 | 223 | 10 | 4(4) |
| 103 | Multiple Sugar-Binding Transport Atp-Binding Protein Msmk | L. monocytogenes FSL F2-208 | 34636 | 5.97 | 216 | 19 | 3(3) |
| 104 | Pyruvate Dehydrogenase Complex, E1 Component, Pyruvate Dehydrogenase Alpha Subunit | L. monocytogenes str. 4b H7858 | 36682 | 5.65 | 162 | 11 | 4(4) |
| 105 | Pyruvate Dehydrogenase Complex, E1 Component, Pyruvate Dehydrogenase Alpha Subunit | L. monocytogenes str. 4b H7858 | 36682 | 5.65 | 132 | 11 | 3(3) |
| 106 | Pyruvate Dehydrogenase Complex, E1 Component, Pyruvate Dehydrogenase Alpha Subunit | L. monocytogenes str. 4b H7858 | 36682 | 5.65 | 192 | 11 | 4(4) |
| 107 | Nadph Dehydrogenase | L. monocytogenes serotype 4b str. LL195 | 38368 | 8.30 | 140 | 10 | 3(3) |
| 108 | Multispecies: Glyceraldehyde-3-Phosphate Dehydrogenase | L. | 36421 | 5.12 | 131 | 15 | 3(3) |
| 109 | Cell Shape Determining Protein Mreb | L. monocytogenes FSL R2-503 | 36988 | 5.23 | 158 | 10 | 3(3) |
| 110 | Gap, Partial | L. innocua | 22226 | 5.25 | 98 | 18 | 2(2) |
| 111 | Aspartate-Semialdehyde Dehydrogenase | L. monocytogenes FSL R2-503 | 25748 | 6.34 | 42 | 15 | 3(3) |
| 112 | Glutamyl Aminopeptidase | L. monocytogenes FSL F2-208 | 38709 | 5.69 | 289 | 12 | 3(3) |
| 113 | Phosphate Acetyltransferase | L. monocytogenes | 15308 | 6.85 | 47 | 17 | 3(3) |
| 115 | Bifunctional Protein Fold | L. monocytogenes | 31002 | 5.44 | 54 | 15 | 2(2) |
| 116 | Conserved Hypothetical Protein | L. monocytogenes serotype 1/2a str. F6854 | 36925 | 5.36 | 111 | 17 | 2(2) |
| 117 | Glutamyl Aminopeptidase | L. monocytogenes FSL F2-208 | 38709 | 5.69 | 174 | 18 | 2(2) |
| 118 | Fructokinase | L. monocytogenes FSL F2-208 | 31579 | 5.65 | 161 | 12 | 3(3) |
| 120 | Flagellin | L. monocytogenes | 30409 | 4.91 | 134 | 9 | 2(2) |
| 121 | Oxidoreductase, Aldo/Keto Reductase Family | L. monocytogenes str. 4b H7858 | 31717 | 4.87 | 86 | 8 | 2(2) |
|  | Nad+ Synthetase | L. monocytogenes FSL F2-208 | 30626 | 5.00 | 68 | 4 | 2(2) |
| 122 | Oxidoreductase, Aldo/Keto Reductase Family | L. monocytogenes str. 4b H7858 | 31717 | 4.87 | 203 | 16 | 4(4) |
| 124 | 6-Phosphofructokinase | L. monocytogenes str. 4b H7858 | 27165 | 5.40 | 65 | 11 | 2(2) |
| 127 | Utp-Glucose-1-Phosphate Uridylyltransferase | L. monocytogenes F6900 | 37183 | 5.45 | 113 | 15 | 2(2) |
| 131 | Gtp-Sensing Transcriptional Pleiotropic Repressor Cody | L. monocytogenes FSL F2-208 | 28495 | 4.90 | 97 | 10 | 2(2) |
| 133 | Gntr Family Transcriptional Regulator | L. monocytogenes | 28008 | 5.13 | 71 | 12 | 2(2) |
| 134 | Methionine Aminopeptidase, Type I | L. monocytogenes FSL F2-208 | 22426 | 5.21 | 39 | 16 | 3(3) |
| 140 | Multispecies: Nad Kinase 2 | L. | 30594 | 6.54 | 46 | 14 | 3(3) |
| 141 | 50s Ribosomal Protein L25 | L. monocytogenes | 22641 | 4.44 | 171 | 28 | 5(5) |
| 144 | 2,3,4,5-Tetrahydropyridine-2-Carboxylate N-Succinyltransferase, Putative | L. monocytogenes str. 4b H7858 | 10942 | 5.74 | 50 | 12 | 3(3) |
| 145 | Naphthoate Synthase | L. monocytogenes HPB2262 | 27681 | 5.72 | 233 | 19 | 3(3) |
| 147 | Coa-Binding Domain Protein | L. monocytogenes str. 4b H7858 | 19366 | 5.56 | 32 | 12 | 3(3) |
| 150 | Adenylate Kinase | L. monocytogenes str. 4b H7858 | 23804 | 5.00 | 43 | 14 | 2(2) |
| 152 | Adenylate Kinase | L. monocytogenes str. 4b H7858 | 23804 | 5.00 | 88 | 13 | 2(2) |
| 154 | 2,3-Bisphosphoglycerate-Dependent Phosphoglycerate Mutase | L. monocytogenes serotype 4b str. LL195 | 27629 | 5.62 | 85 | 16 | 2(2) |
| 156 | Chain A, Crystal Structure Of 3-Oxoacyl-[Acyl-Carrier Protein]Reductase (Fabg) From Listeria Monocytogenes In Complex With Nadp+ |  | 29025 | 5.82 | 86 | 10 | 2(2) |
| 158 | Transcriptional Regulatory Protein Degu | L. monocytogenes FSL F2-208 | 14527 | 9.24 | 41 | 19 | 2(2) |
| 159 | Ribosomal Protein S3 | L. monocytogenes str. 1/2a F6854 | 17247 | 9.89 | 31 | 19 | 2(2) |
| 162 | Phosphoglycerate Kinase/Triose-Phosphate Isomerase | L. monocytogenes FSL N3-165 | 27608 | 4.78 | 80 | 13 | 2(2) |
| 165 | Uracil Phosphoribosyltransferase | L. monocytogenes FSL R2-561 | 16944 | 5.70 | 33 | 10 | 2(2) |
| 166 | Abc Transporter, Atp-Binding Protein | L. monocytogenes str. 4b H7858 | 12617 | 4.97 | 67 | 31 | 2(2) |
| 176 | Ribosome-Recycling Factor | L. monocytogenes | 20743 | 5.25 | 40 | 12 | 2(2) |
| 178 | Uracil Phosphoribosyltransferase | L. monocytogenes FSL R2-561 | 16944 | 5.70 | 88 | 18 | 2(2) |
| 184 | Sod | L. monocytogenes | 14658 | 4.95 | 34 | 12 | 2(2) |
| 185 | Sod | L. monocytogenes | 14658 | 4.95 | 79 | 12 | 2(2) |
| 186 | Short-Chain Dehydrogenase | L. monocytogenes | 20932 | 5.91 | 30 | 19 | 2(2) |
| 195 | 50s Ribosomal Protein L5 | L. monocytogenes serotype 4b str. LL195 | 21249 | 9.17 | 84 | 13 | 2(2) |
| 197 | Transcription Elongation Factor Grea | L. monocytogenes str. 4b H7858 | 16570 | 4.58 | 67 | 15 | 2(2) |
| 202 | Pts System Mannose-Specific Eiiab Component | L. monocytogenes serotype 4b str. LL195 | 19655 | 9.33 | 50 | 17 | 2(2) |
| 204 | Putative Universal Stress Protein | L. monocytogenes FSL F2-208 | 17555 | 4.98 | 83 | 16 | 2(2) |
| 212 | Multispecies: 30s Ribosomal Protein S8 | L. | 14635 | 9.48 | 71 | 19 | 2(2) |
| 213 | Hypothetical Protein | L. monocytogenes | 11250 | 4.46 | 38 | 10 | 2(2) |
| 218 | Ribosomal Protein L31 | L. monocytogenes | 9241 | 8.93 | 36 | 13 | 2(2) |
| 219 | 50s Ribosomal Protein L7/L12 | L. monocytogenes | 12462 | 4.54 | 47 | 10 | 2(2) |
| 231 | Dna-Binding Protein Hu | L. monocytogenes serotype 4b str. LL195 | 13590 | 9.10 | 32 | 12 | 2(2) |
| 233 | Dna-Binding Protein Hu | L. monocytogenes serotype 4b str. LL195 | 13590 | 9.10 | 90 | 22 | 2(2) |
| 248 | Purine Nucleoside Phosphorylase | L. monocytogenes | 25341 | 4.87 | 39 | 16 | 2(2) |
| 253 | GroES | L. monocytogenes | 10042 | 4.60 | 31 | 15 | 2(2) |
| B ot100 | Multispecies: Enolase | L. | 46458 | 4.70 | 84 | 7 | 2(2) |
| E Eot100 | Conserved Hypothetical Protein | L. monocytogenes str. 4b H7858 | 11284 | 4.79 | 39 | 14 | 2(2) |
| F ot100 | 30s Ribosomal Protein S2 | L. monocytogenes serotype 4b str. LL195 | 30581 | 6.28 | 38 | 13 | 2(2) |
|  |  |  |  |  |  |  |  |
