# Supplementary material for: How Listeria monocytogenes Shapes Its Proteome in Response to Natural Antimicrobial Compounds
Source: Front Microbiol. 2019 Mar 12;10:437. doi: 10.3389/fmicb.2019.00437 (PMC6423498; doi:10.3389/fmicb.2019.00437)
Supplement: Supplementary file 3 [file Data_Sheet_1.PDF]

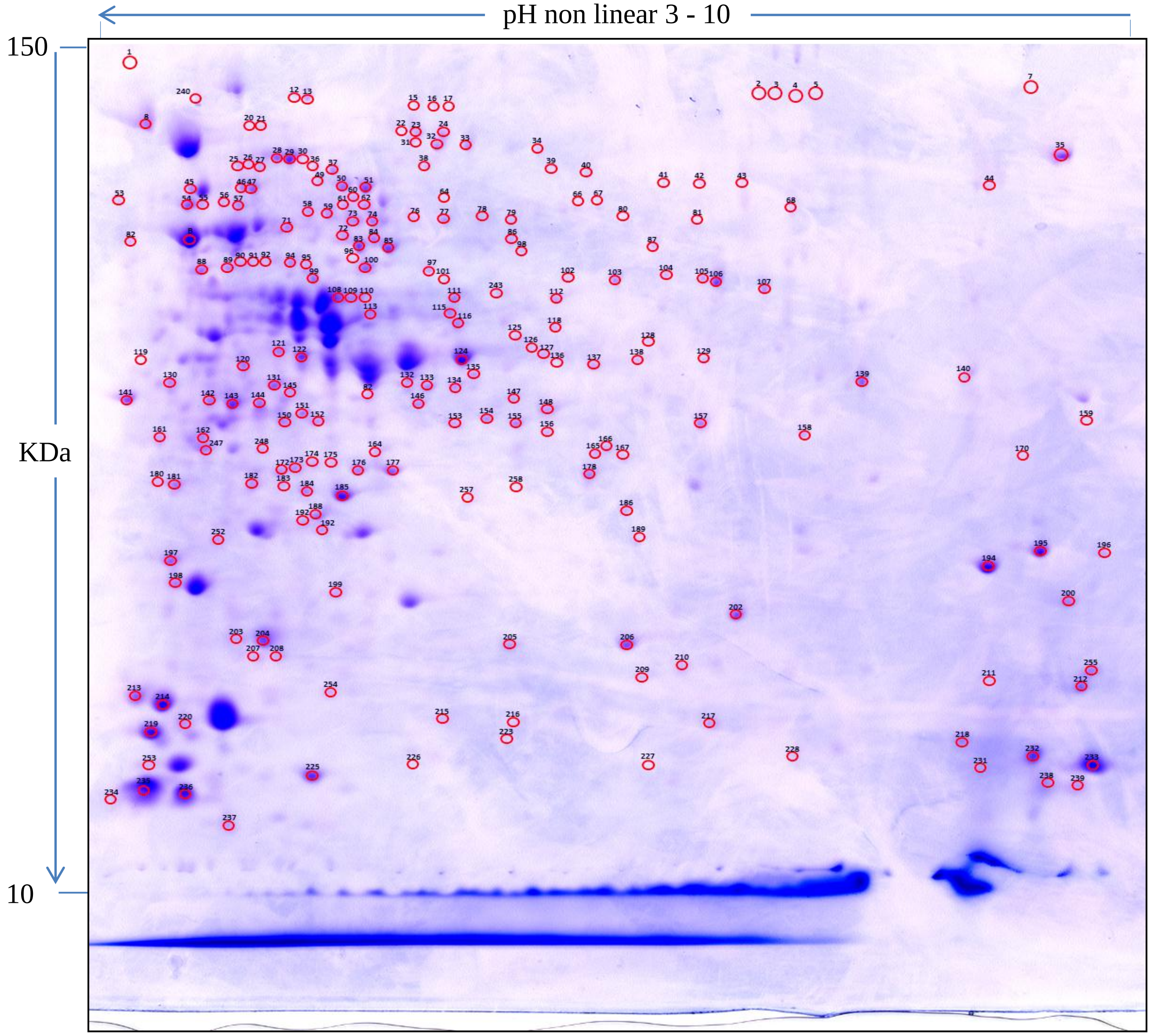

Supplementary Figure 1. Two-dimensional electrophoresis analysis of intracellular proteins synthesized by *Listeria monocytogenes* Scott A cells grown on BHI broth until the middle exponential phase of growth (OD= 0.4, λ=600 nm) was reached (untreated cells). The numbered circles refer to proteins with decreased or increased amount during treatment with antimicrobial compounds. Spot designation corresponds to that of the proteins in Supplementary Table 1.

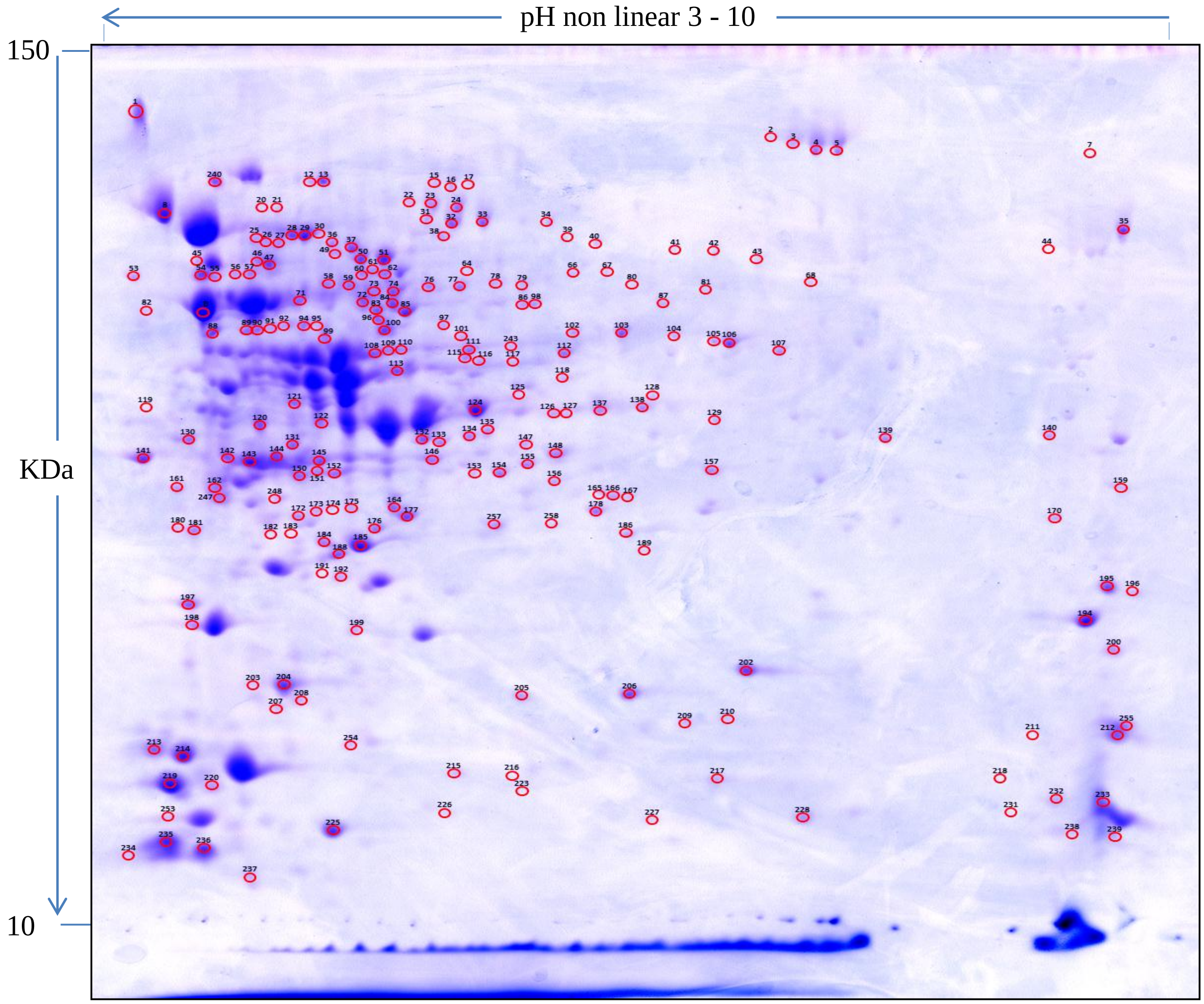

Supplementary Figure 2. Two-dimensional electrophoresis analysis of intracellular proteins synthesized by *Listeria monocytogenes* Scott A cells grown on BHI broth until the middle exponential phase of growth (OD= 0.4, λ=600 nm) was reached and treated for one hour to ethanol (1% v/v). The numbered circles refer to proteins with decreased or increased amount compared to un-treated cells. Spot designation corresponds to that of the proteins in Supplementary Table 1.

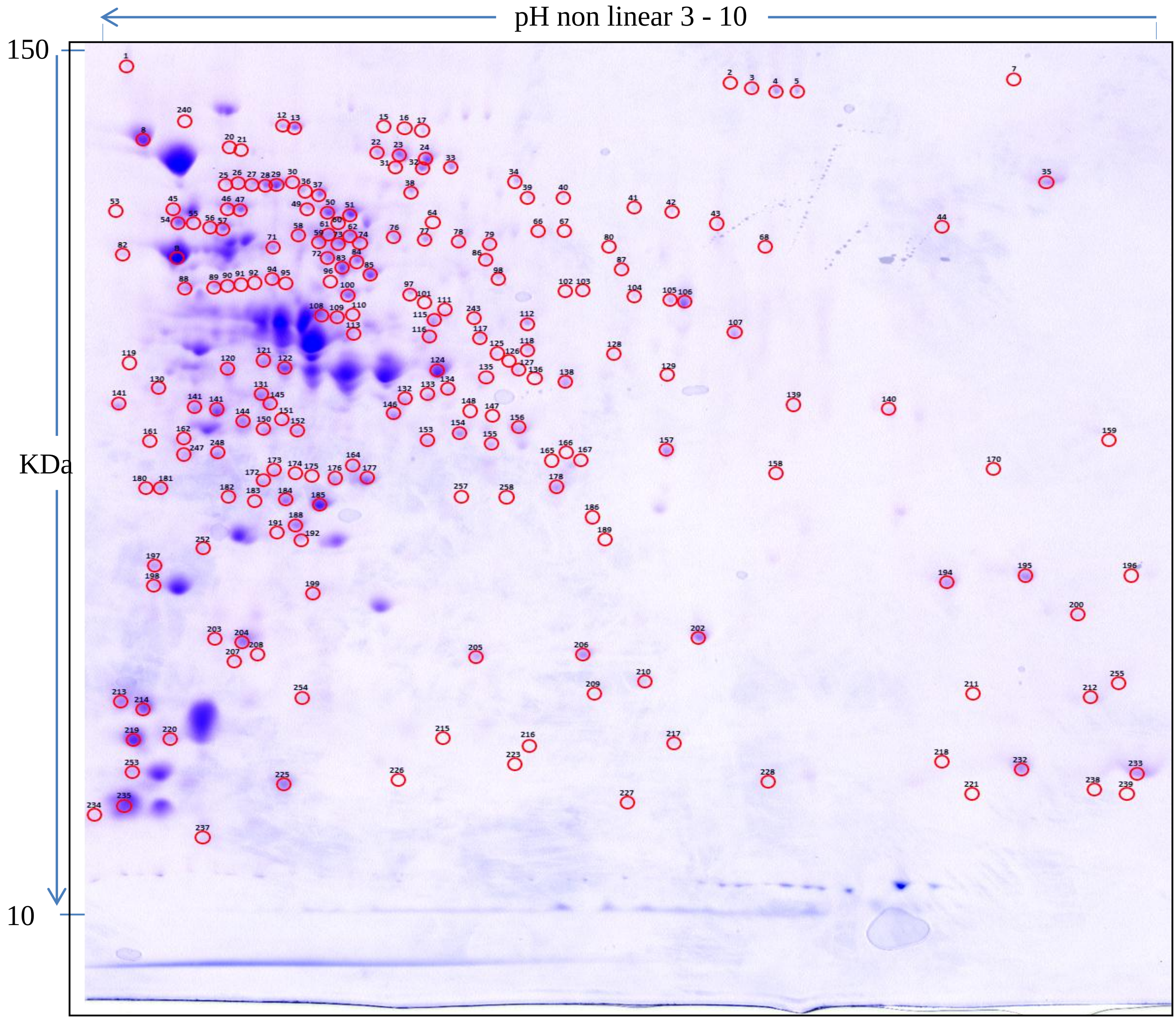

Supplementary Figure 3A. Two-dimensional electrophoresis analysis of intracellular proteins synthesized by *Listeria monocytogenes* Scott A cells grown on BHI broth until the middle exponential phase of growth (OD= 0.4, λ=600 nm) was reached and treated for one hour to ethanol (1% v/v) and citral at 85 mg/L. The numbered circles refer to proteins with decreased or increased amount compared to un-treated cells. Spot designation corresponds to that of the proteins in Supplementary Table 1.

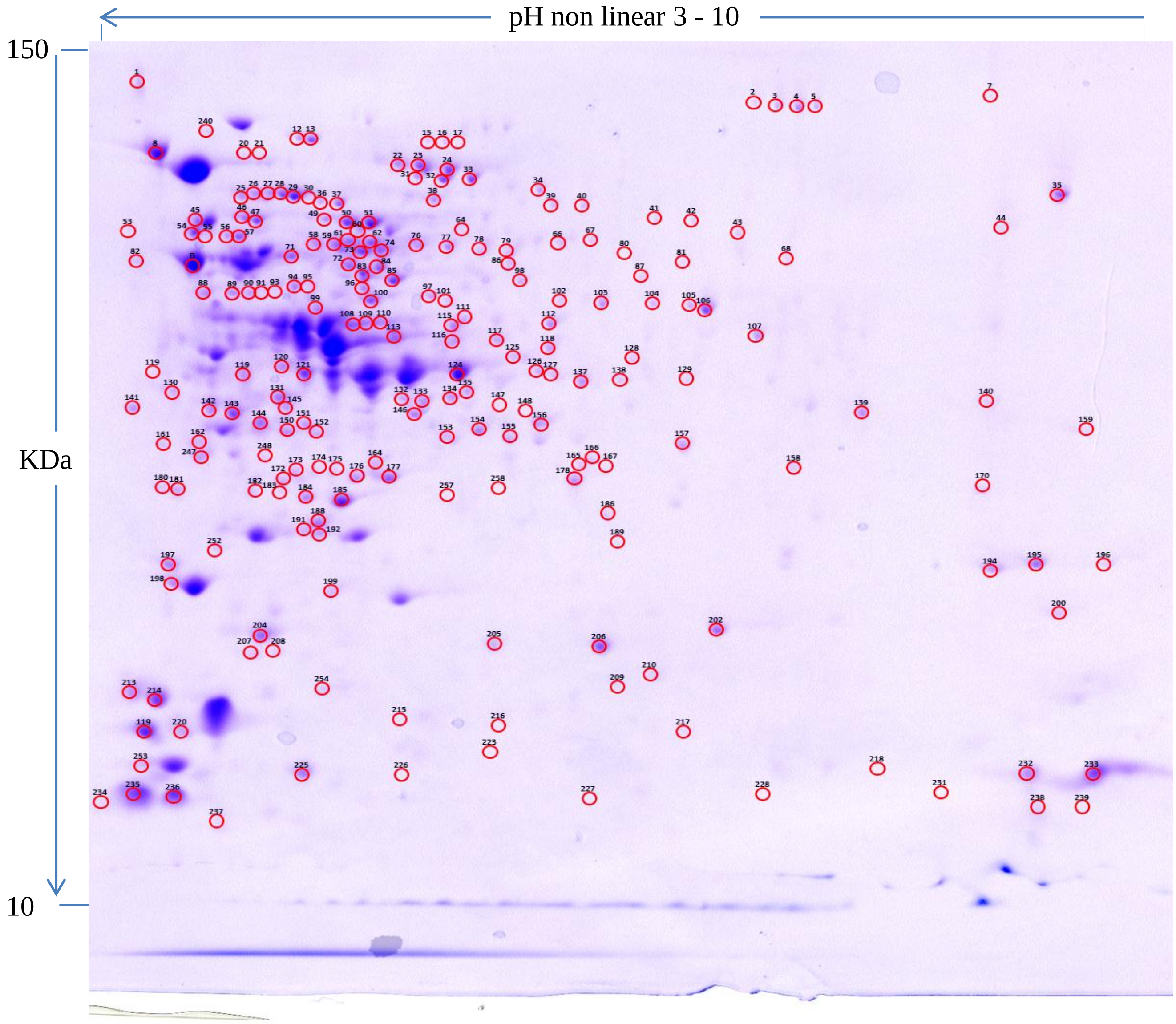

Supplementary Figure 3B. Two-dimensional electrophoresis analysis of intracellular proteins synthesized by *Listeria monocytogenes* Scott A cells grown on BHI broth until the middle exponential phase of growth (OD= 0.4, λ=600 nm) was reached and treated for one hour to ethanol (1% v/v) and citral at 125 mg/L. The numbered circles refer to proteins with decreased or increased amount compared to un-treated cells. Spot designation corresponds to that of the proteins in Supplementary Table 1.

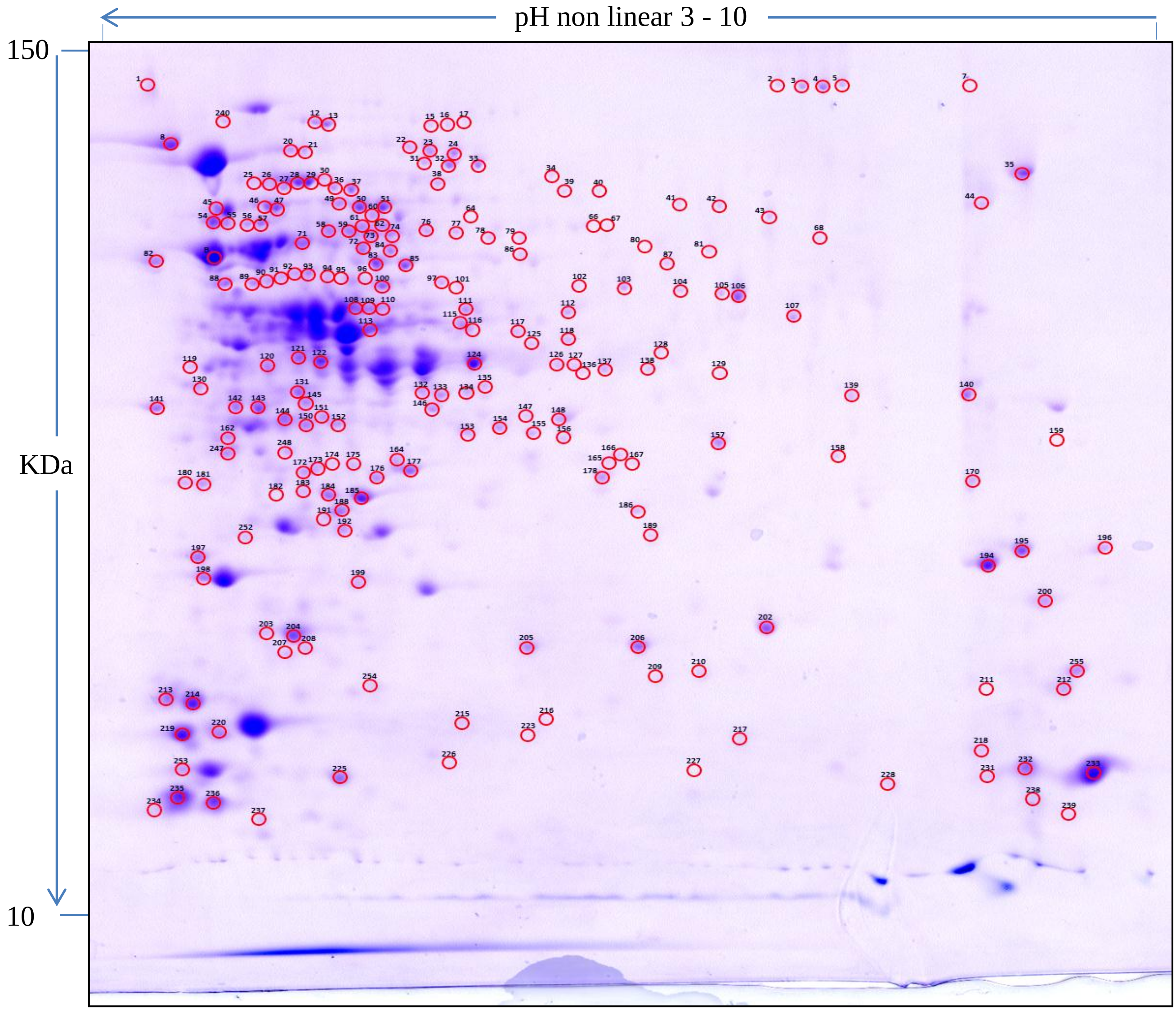

Supplementary Figure 4A. Two-dimensional electrophoresis analysis of intracellular proteins synthesized by *Listeria monocytogenes* Scott A cells grown on BHI broth until the middle exponential phase of growth (OD= 0.4, λ=600 nm) was reached and treated for one hour to ethanol (1% v/v) and carvacrol at 20 mg/L. The numbered circles refer to proteins with decreased or increased amount compared to un-treated cells. Spot designation corresponds to that of the proteins in Supplementary Table 1.

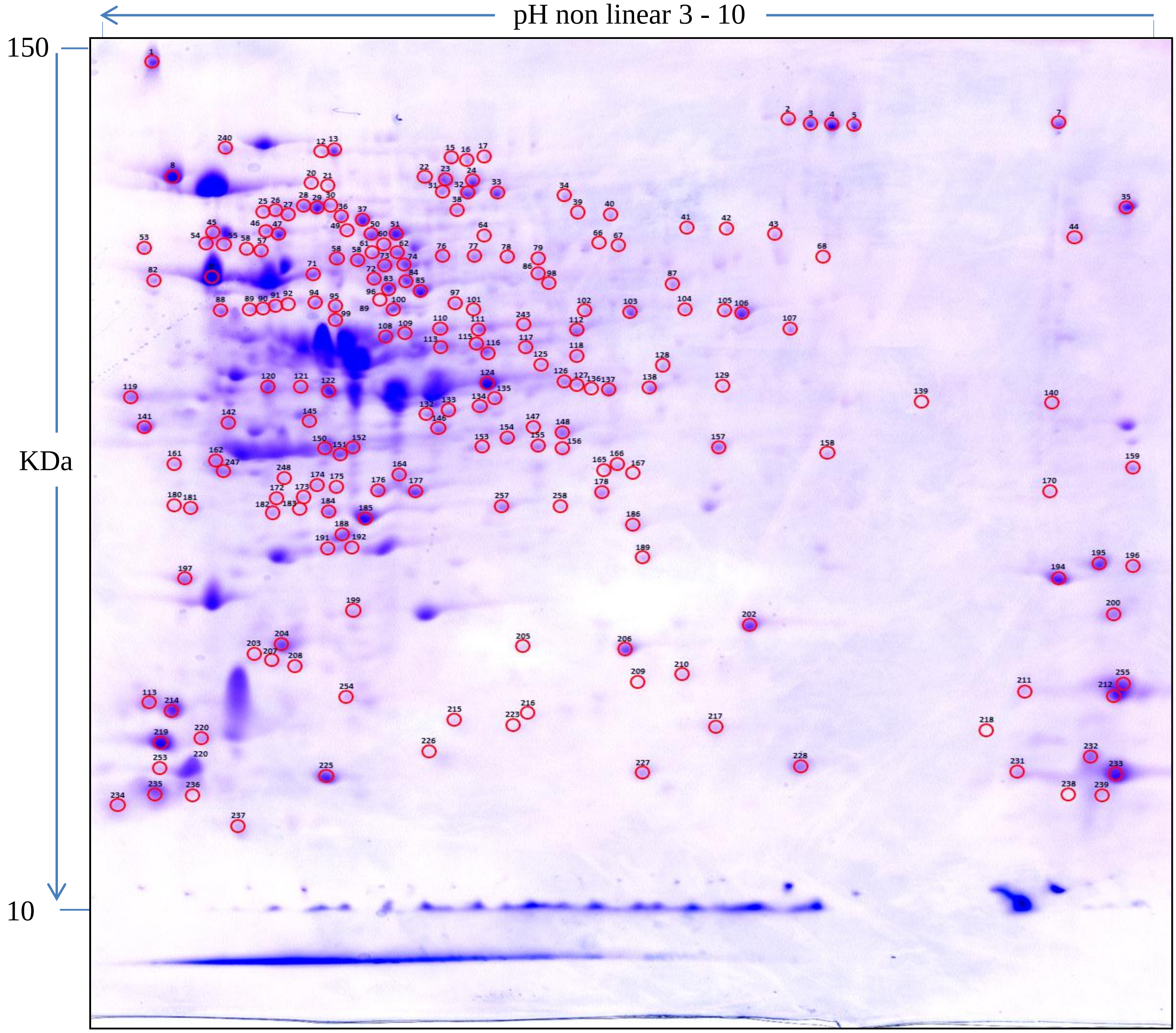

Supplementary Figure 4B. Two-dimensional electrophoresis analysis of intracellular proteins synthesized by *Listeria monocytogenes* Scott A cells grown on BHI broth until the middle exponential phase of growth (OD= 0.4, λ=600 nm) was reached and treated for one hour to ethanol (1% v/v) and and carvacrol at 35 mg/L. The numbered circles refer to proteins with decreased or increased amount compared to un-treated cells. Spot designation corresponds to that of the proteins in Supplementary Table 1.

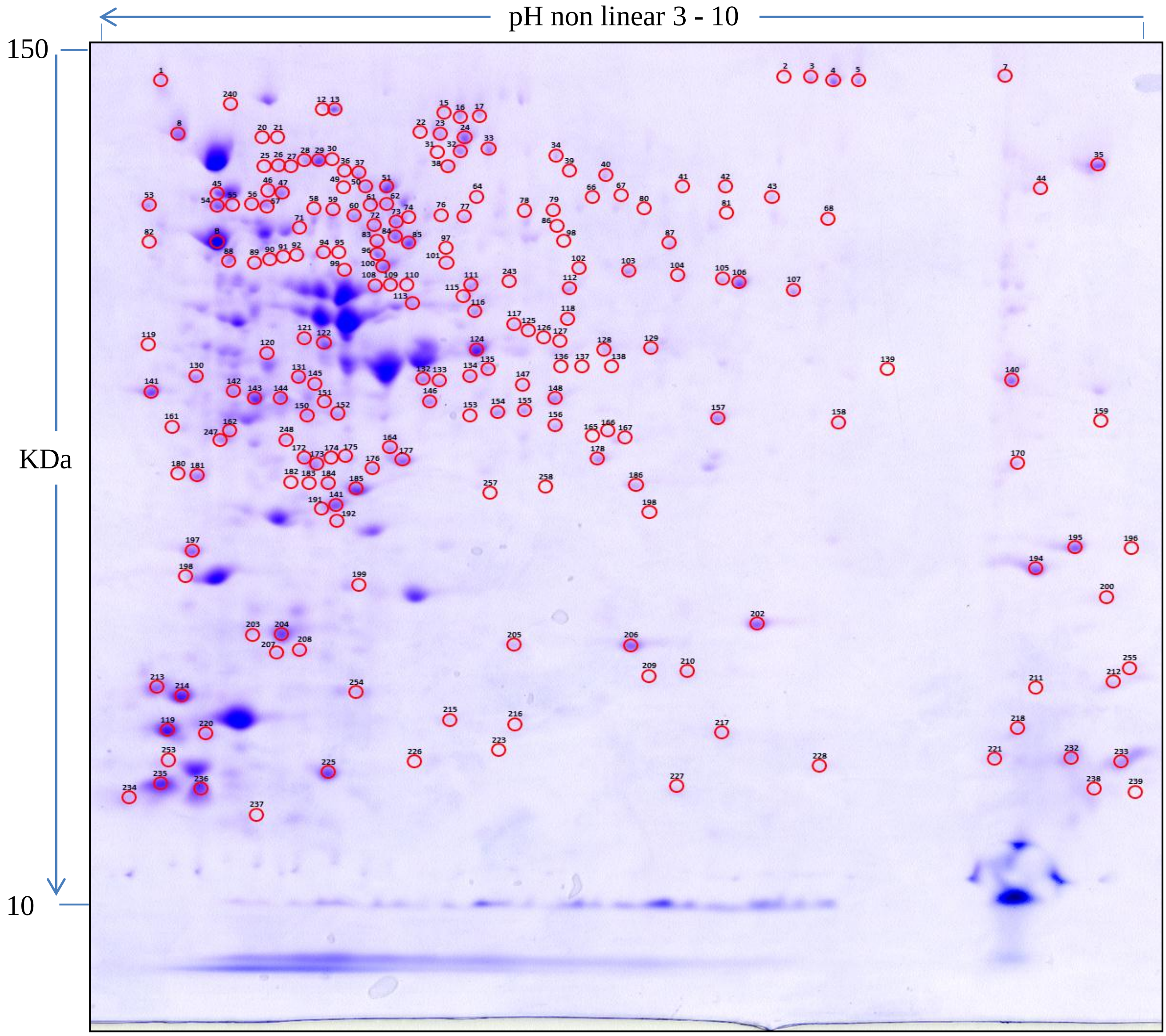

Supplementary Figure 4C. Two-dimensional electrophoresis analysis of intracellular proteins synthesized by *Listeria monocytogenes* Scott A cells grown on BHI broth until the middle exponential phase of growth (OD= 0.4, λ=600 nm) was reached and treated for one hour to ethanol (1% v/v) and carvacrol at 50 mg/L. The numbered circles refer to proteins with decreased or increased amount compared to un-treated cells. Spot designation corresponds to that of the proteins in Supplementary Table 1.

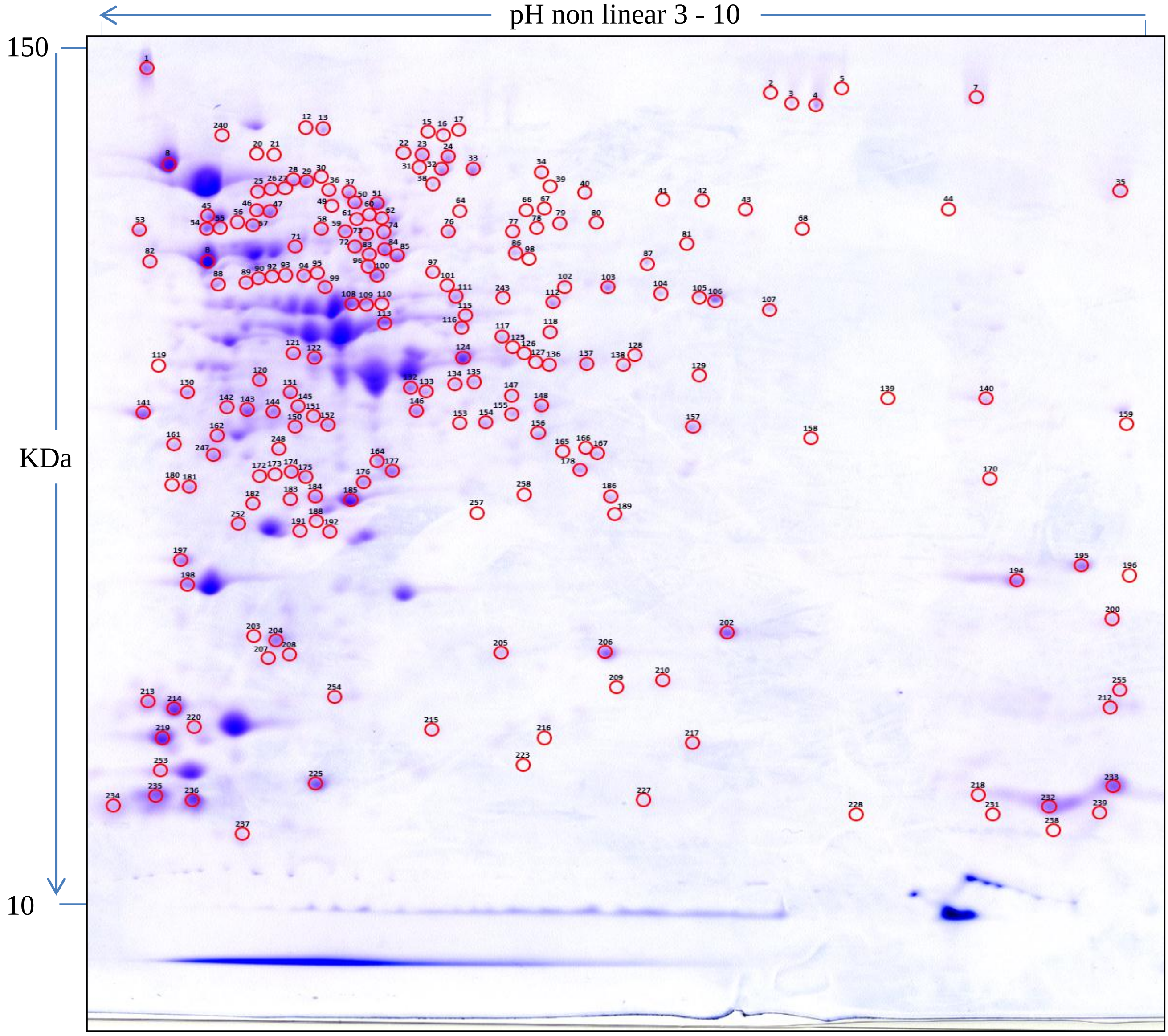

Supplementary Figure 5A. Two-dimensional electrophoresis analysis of intracellular proteins synthesized by *Listeria monocytogenes* Scott A cells grown on BHI broth until the middle exponential phase of growth (OD= 0.4, λ=600 nm) was reached and treated for one hour to ethanol (1% v/v) and (E)-2-hexenal at 150 mg/L. The numbered circles refer to proteins with decreased or increased amount compared to un-treated cells. Spot designation corresponds to that of the proteins in Supplementary Table 1.

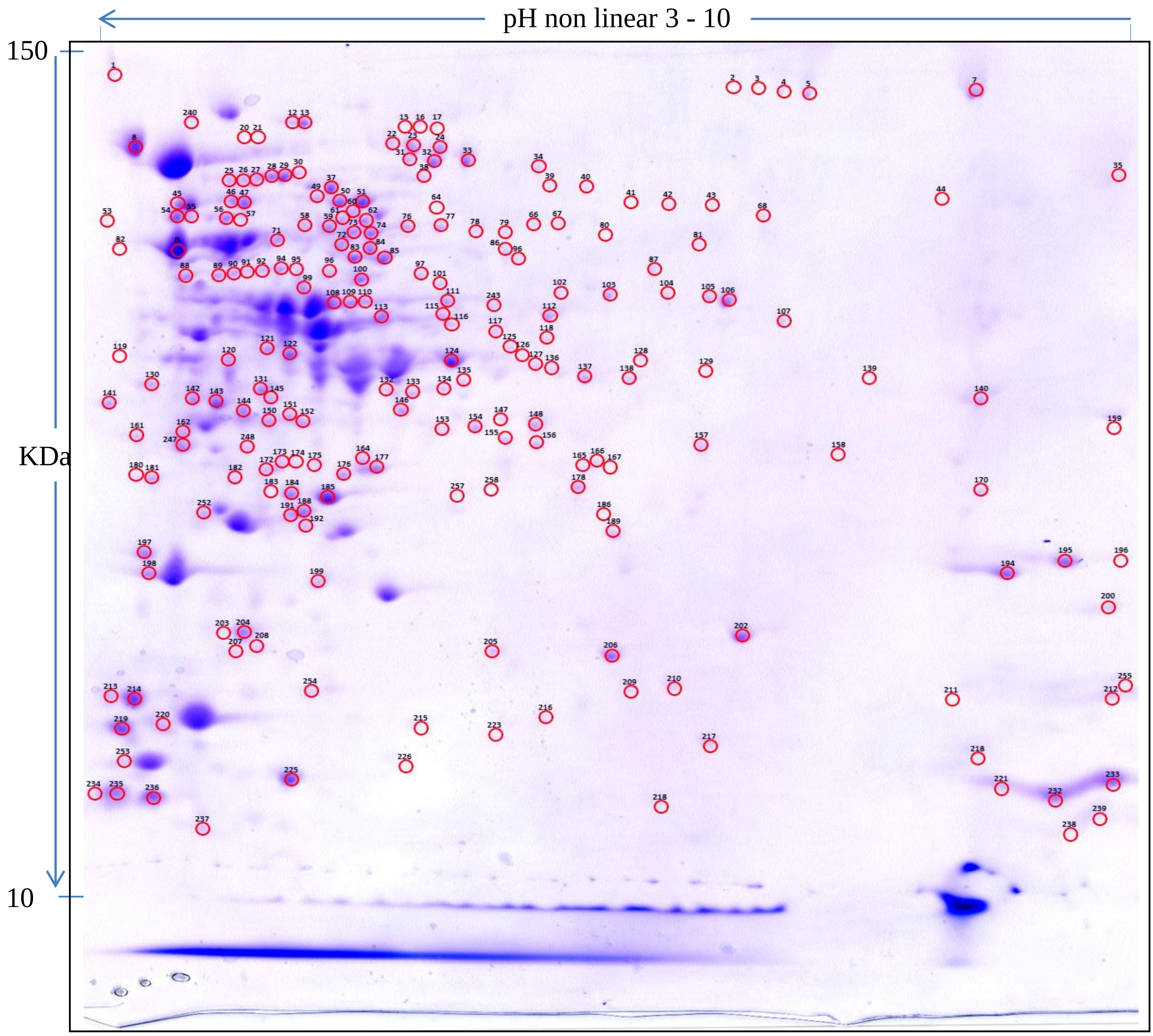

Supplementary Figure 5B. Two-dimensional electrophoresis analysis of intracellular proteins synthesized by *Listeria monocytogenes* Scott A cells grown on BHI broth until the middle exponential phase of growth (OD= 0.4, λ=600 nm) was reached and treated for one hour to ethanol (1% v/v) and (E)-2-hexenal at 250 mg/L. The numbered circles refer to proteins with decreased or increased amount compared to un-treated cells. Spot designation corresponds to that of the proteins in Supplementary Table 1.

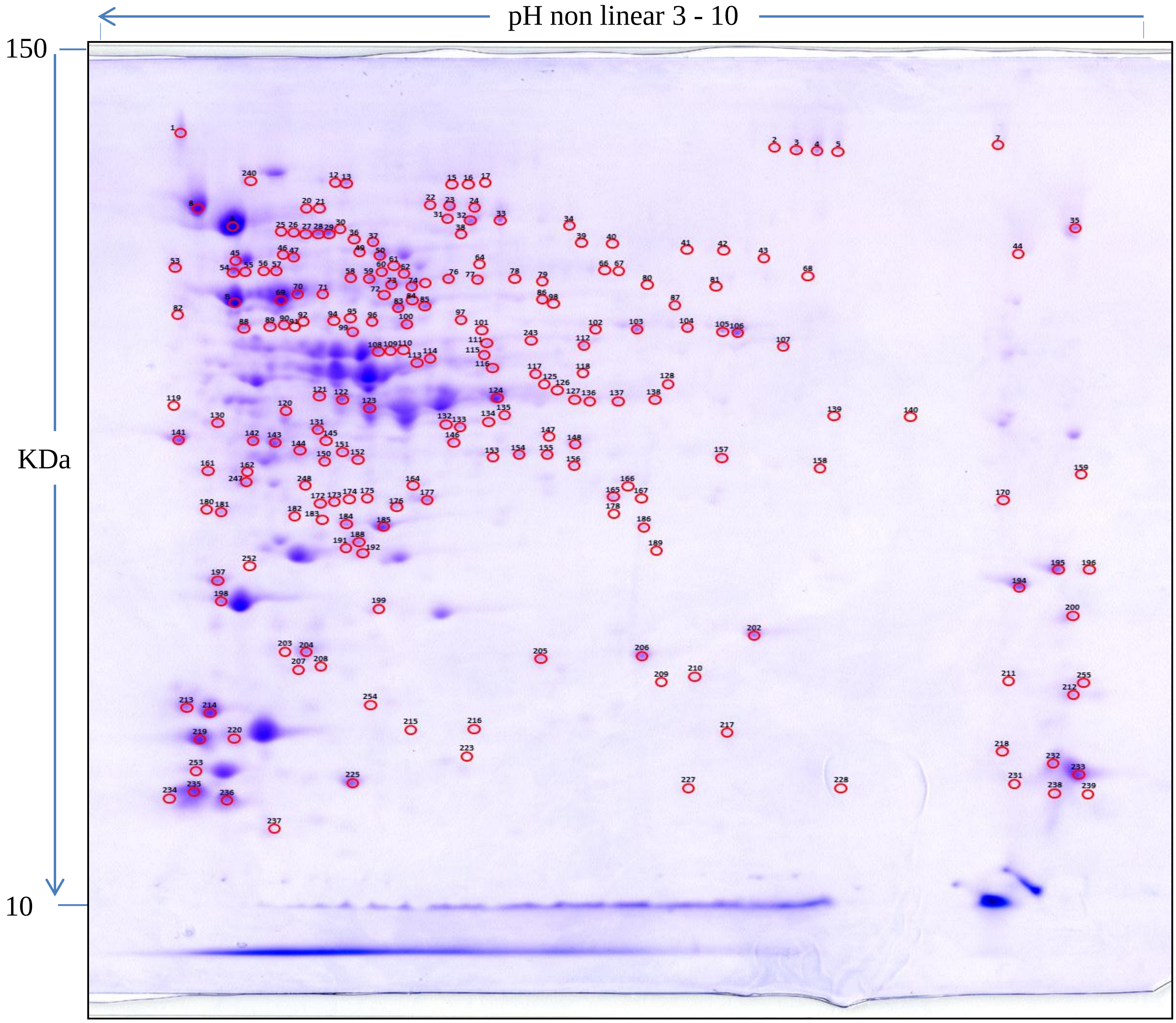

Supplementary Figure 5C. Two-dimensional electrophoresis analysis of intracellular proteins synthesized by *Listeria monocytogenes* Scott A cells grown on BHI broth until the middle exponential phase of growth (OD= 0.4, λ=600 nm) was reached and treated for one hour to ethanol (1% v/v) and (E)-2-hexenal at 400 mg/L. The numbered circles refer to proteins with decreased or increased amount compared to un-treated cells. Spot designation corresponds to that of the proteins in Supplementary Table 1.

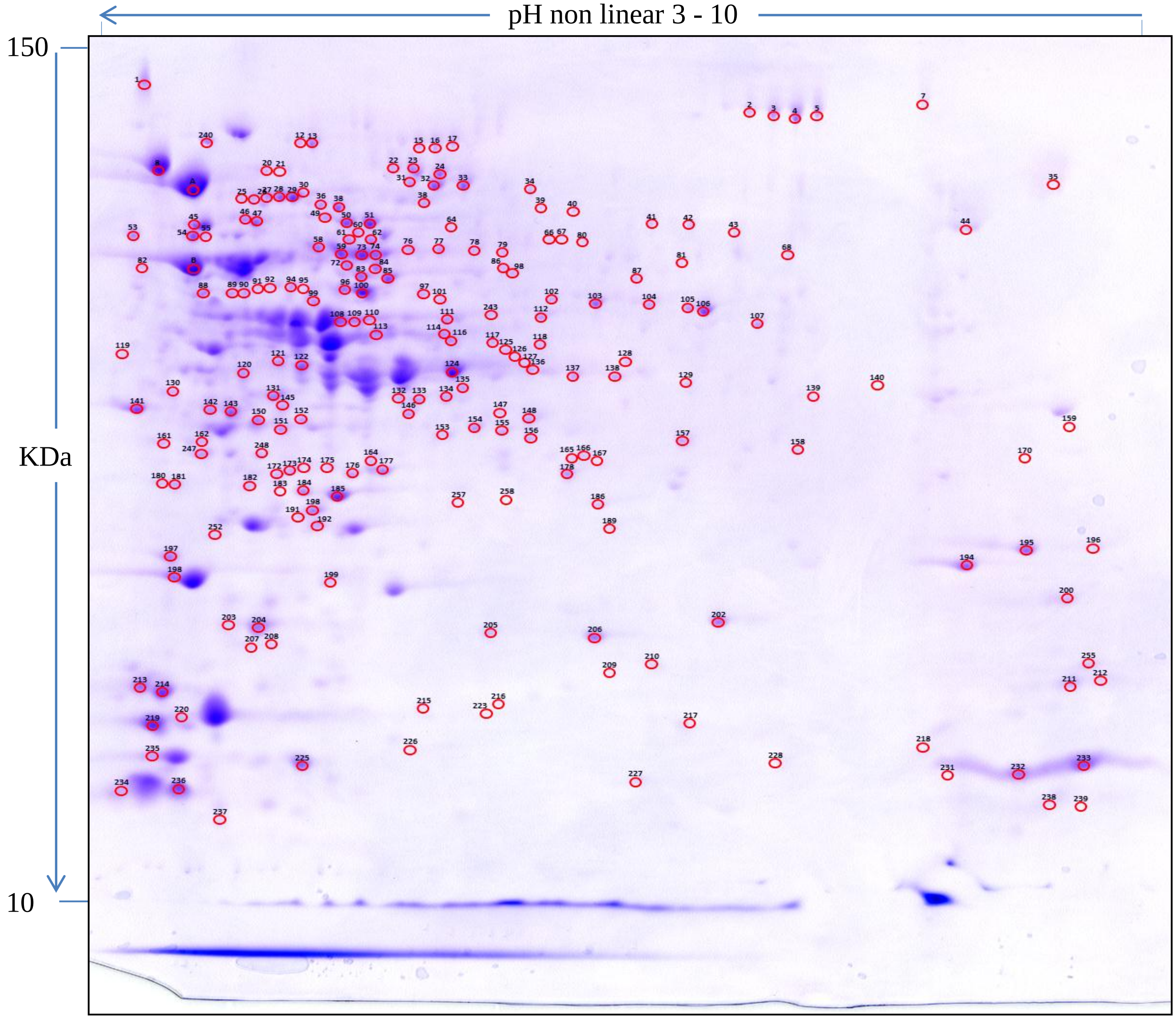

Supplementary Figure 6A. Two-dimensional electrophoresis analysis of intracellular proteins synthesized by *Listeria monocytogenes* Scott A cells grown on BHI broth until the middle exponential phase of growth (OD= 0.4, λ=600 nm) was reached and treated for one hour to ethanol (1% v/v) and Thyme EO at 40 mg/L. The numbered circles refer to proteins with decreased or increased amount compared to un-treated cells. Spot designation corresponds to that of the proteins in Supplementary Table 1.

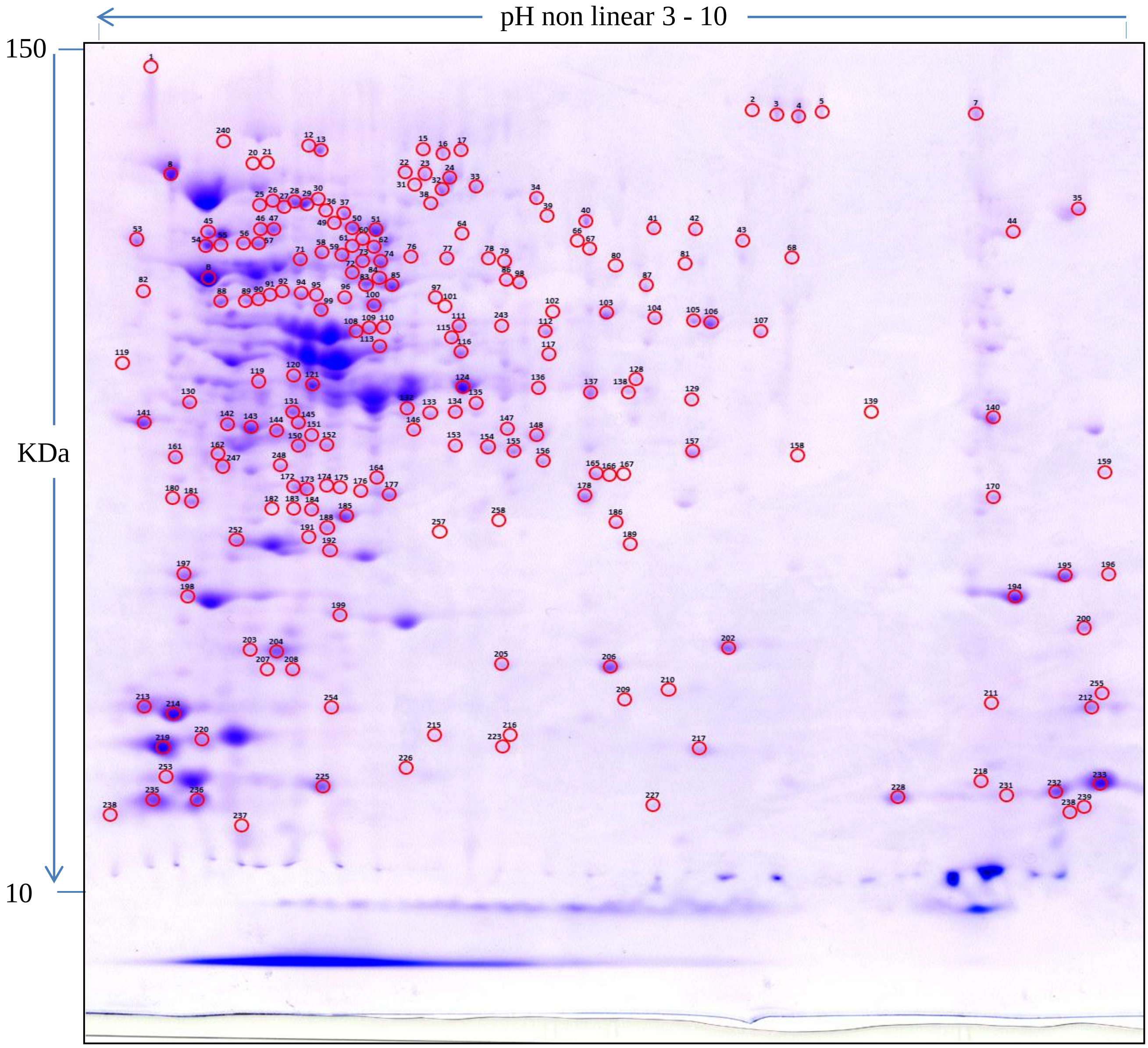

Supplementary Figure 6B. Two-dimensional electrophoresis analysis of intracellular proteins synthesized by *Listeria monocytogenes* Scott A cells grown on BHI broth until the middle exponential phase of growth (OD= 0.4, λ=600 nm) was reached and treated for one hour to ethanol (1% v/v) and Thyme EO at 70 mg/L. The numbered circles refer to proteins with decreased or increased amount compared to un-treated cells. Spot designation corresponds to that of the proteins in Supplementary Table 1.

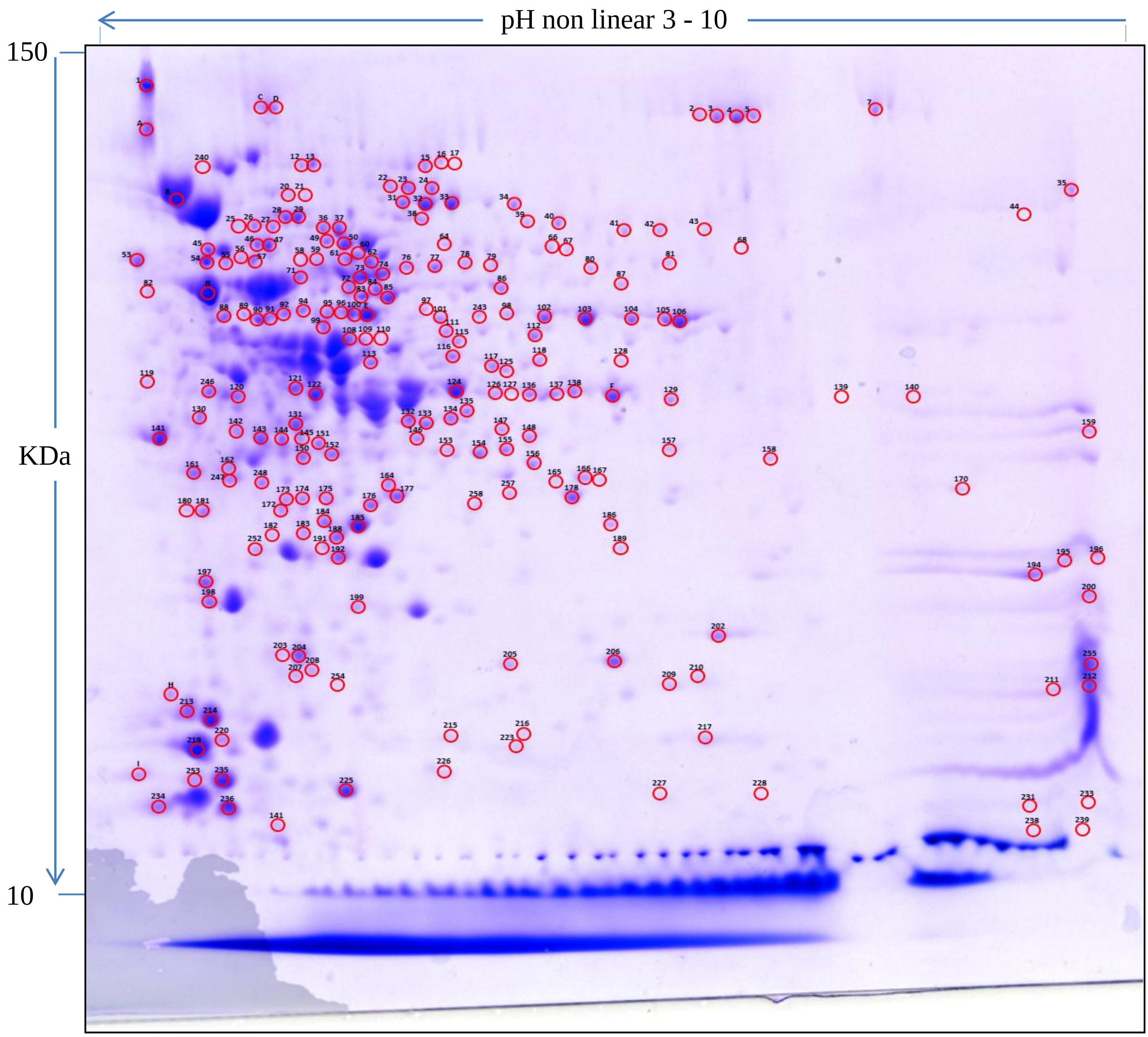

Supplementary Figure 6C. Two-dimensional electrophoresis analysis of intracellular proteins synthesized by *Listeria monocytogenes* Scott A cells grown on BHI broth until the middle exponential phase of growth (OD= 0.4, λ=600 nm) was reached and treated for one hour to ethanol (1% v/v) and Thyme EO at 100 mg/L. The numbered circles refer to proteins with decreased or increased amount compared to un-treated cells. Spot designation corresponds to that of the proteins in Supplementary Table 1.

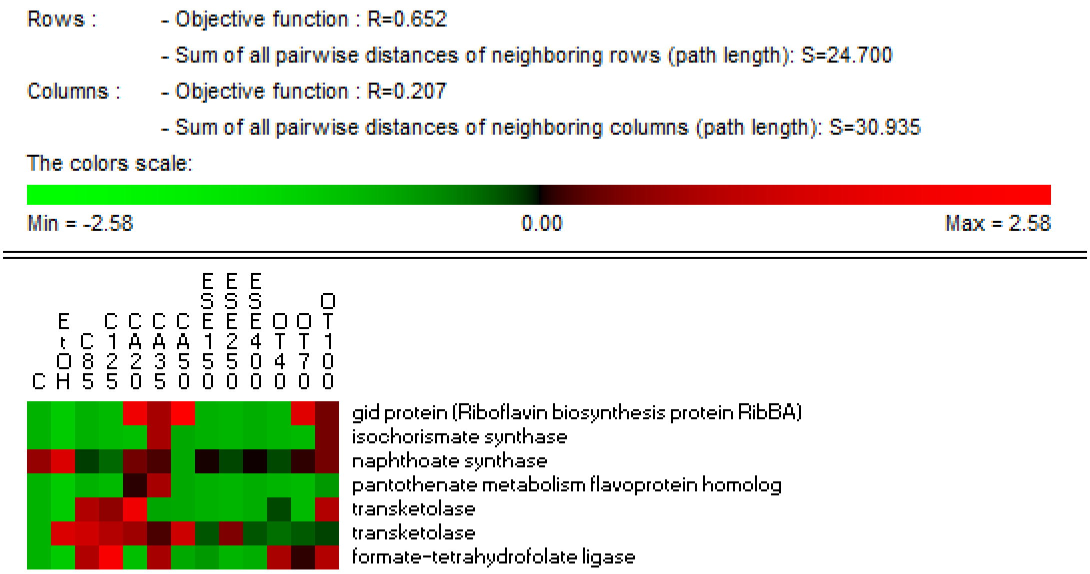

Supplementary Figure 7. Heat map of the identified proteins involved in vitamin and cofactor metabolism in cells of *Listeria monocytogenes* Scott A. Only protein spots showing different (≥ or ≤ of 2 fold, P<0.05) relative amounts under stress conditions compared to untreated cells were showed. C, untreated cells; EtOH, cells treated with ethanol (1% v/v) alone. Other antimicrobial compounds were: citral at 85 (C85) and 125 (C125) mg/L; carvacrol at 20 (CA20), 35 (CA35) or 50 (CA50); (E)-2-hexenal at 150 (ESE1), 250 (ESE2) or 400 (ESE4) mg/L; and thyme essential oil at 40 (OT40), 70 (OT70) or 100 (OT100) mg/L.

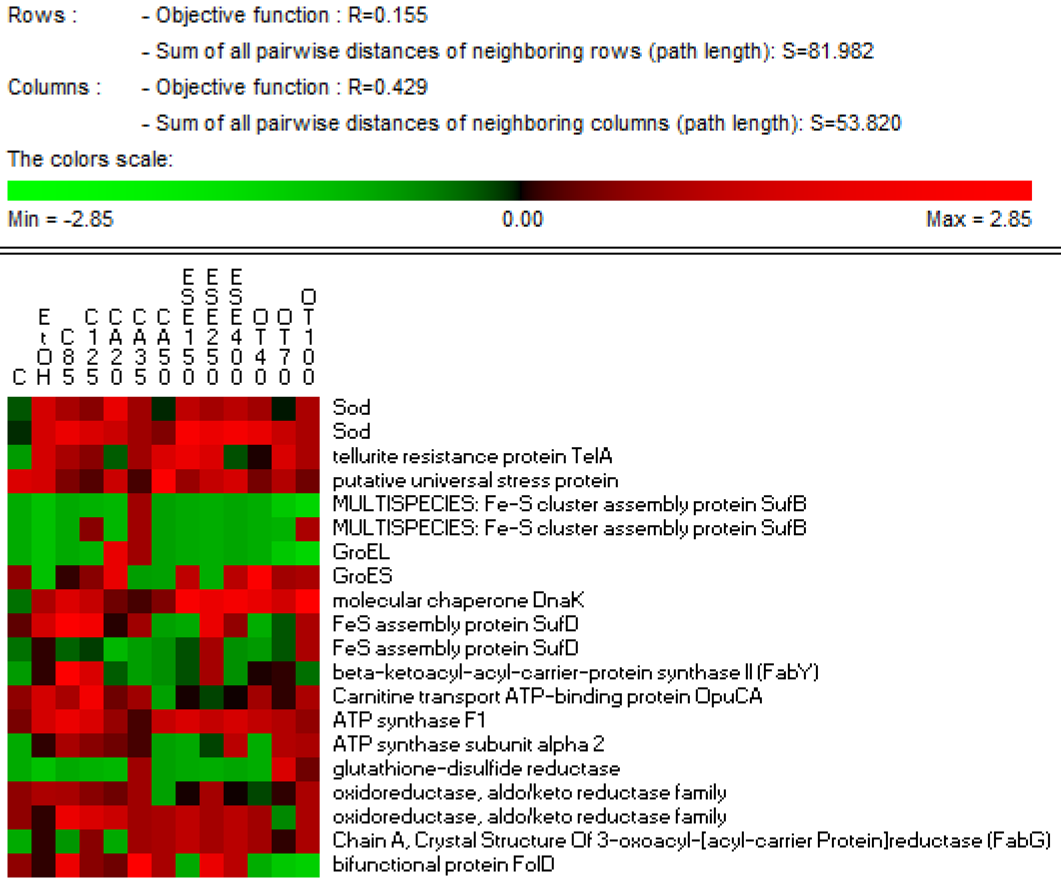

Supplementary Figure 8. Heat map of the identified proteins involved in stress resistance in cells of *Listeria monocytogenes* Scott A. Only protein spots showing different (≥ or ≤ of 2 fold, P<0.05) relative amounts under stress conditions compared to untreated cells were showed. C, untreated cells; EtOH, cells treated with ethanol (1% v/v) alone. Other antimicrobial compounds were: citral at 85 (C85) and 125 (C125) mg/L; carvacrol at 20 (CA20), 35 (CA35) or 50 (CA50); (E)-2-hexenal at 150 (ESE1), 250 (ESE2) or 400 (ESE4) mg/L; and thyme essential oil at 40 (OT40), 70 (OT70) or 100 (OT100) mg/L.
